# Stigmurin derivatives as potent-biofilm eradicating agents against the major human opportunistic pathogen *Pseudomonas aeruginosa*

**DOI:** 10.3389/fmicb.2026.1827386

**Published:** 2026-05-28

**Authors:** Anne-Sophie Tareau, Adriana Marina e Silva Parente, Allanny Alves Furtado, Magalie Barreau, Hung Le, Bruno Amorim-Carmo, Lucas Hilário Nogueira de Sousa, Olivier Maillot, Mathieu Gonzalez, Adrien Forge, Ali Tahrioui, Jarbas Magalhaes Resende, Olivier Lesouhaitier, Renata Mendonça Araújo, Matheus de Freitas Fernandes-Pedrosa, Sylvie Chevalier

**Affiliations:** 1 Univ Rouen Normandie, Université Caen Normandie, Normandie Univ, CBSA, UR, Rouen, France; 2 Univ Rouen Normandie, Platform of Sanitary Safety, Evreux Campus, Evreux, France; 3 Biotechnology Laboratory in Biomolecules (LBB), Department of Biochemistry, Center for Biosciences, Federal University of Rio Grande do Norte (UFRN), Natal, RN, Brazil; 4 Laboratory of Isolation and Synthesis of Organic Compounds, Department of Biochemistry, Institute of Chemistry, Federal University of Rio Grande do Norte (UFRN), Natal, RN, Brazil; 5 Laboratório de Síntese e Estrutura de Peptídeos, Departamento de Química, Universidade Federal de Minas Gerais, Belo Horizonte, Brazil

**Keywords:** biofilm eradication, dynamic and static biofilms, NMR, *Pseudomonas aeruginosa*, Stigmurin analogs

## Abstract

*Pseudomonas aeruginosa* is a versatile opportunistic pathogen whose capacity to form biofilms contributes to persistent and difficult-to-treat infections, particularly in clinical environments. Conventional antibiotics often fail to eliminate biofilm-embedded bacteria, highlighting the need for alternative therapeutic strategies. Here, we investigated the anti-biofilm activity of Stigmurin, an antimicrobial peptide identified in the venom of *Tityus stigmurus*, together with six lysine-enriched analogs. The peptides were synthesized and characterized at a physicochemical level, and their effects on bacterial growth, biofilm development, and disruption of pre-established biofilms were evaluated under both static and dynamic conditions. Their influence on pyocyanin production, membrane fluidity, and the tridimensional structure of the most active analog, StigA31, was also analyzed. The peptides revealed no marked impact on bacterial growth or biofilm formation. However, several of them were able to disrupt mature biofilms on polystyrene pegs. Stigmurin (24 h exposition) reduced biofilm biomass by nearly 47%, while StigA31 achieved dispersal levels of up to 35%, even at nanomolar concentrations. Under dynamic flow conditions, both peptides strongly reduced biofilm biovolume and thickness within 2 h and altered matrix exopolysaccharide detection. None of the compounds stimulated pyocyanin production. Stigmurin altered membrane fluidity, whereas StigA31 displayed a distinct mechanism that may be associated with its helical conformation. These findings indicate that Stigmurin analogs, particularly StigA31, promote P. aeruginosa biofilm dispersal through non-bactericidal mechanisms. Their activity at sub-inhibitory concentrations highlights their potential as promising candidates for use in strategies aimed at controlling biofilm-associated infections.

## Introduction

Bacterial biofilms or aggregates are structured communities that are embedded within a self-produced matrix of extracellular polymeric compounds, adhering to both living and non-living surfaces (Flemming et al., 2016; Kragh et al., 2023). Biofilm lifestyle provides a protective environment, enhancing bacterial resistance and tolerance to antibiotics and to the host’s immune responses, making biofilms as a critical factor in the persistence and chronicity of infections (Ciofu et al., 2022). Biofilms are involved in a wide range of chronic infections, including those associated with medical devices, such as catheters and prosthetic joints, as well as tissue-related infections like chronic otitis and cystic fibrosis lung infections (de la Fuente-Núñez et al., 2013). The resilience of biofilms against antimicrobial treatments is due to several factors. The matrix acts as a barrier to antibiotic penetration, while the slow growth rate and altered metabolic state of biofilm-associated cells reduce the efficacy of antibiotics that target actively dividing cells (Uruén et al., 2020). Additionally, biofilms can act as reservoirs for persister cells, dormant forms that withstand antibiotic exposure and can repopulate after treatment cessation (Uruén et al., 2020). The presence of biofilms in chronic infections represents a significant challenge in terms of treatment. Indeed, conventional antibiotic therapies are often ineffective against bacteria-embedded within biofilms, thus requiring the development of novel strategies (Guilhen et al., 2017; Rumbaugh and Sauer, 2020). Biofilm disruption is a promising anti-biofilm strategy that promotes the transition of bacteria from a sessile state to a dispersed one. While sessile bacteria are tolerant to antibiotics because of the protective matrix of biofilms, dispersing the biofilm can re-isolate the bacteria and increase their susceptibility to antibiotics (Chua et al., 2014). This makes biofilm-dispersal agents valuable adjuvants to conventional antibiotics treatments.

*Pseudomonas aeruginosa* is a highly versatile and adaptable Gram-negative opportunistic pathogen, that poses a severe threat in clinical settings, in particular for immunocompromised patients. *P. aeruginosa* indeed is known for its intrinsic and acquired resistance to antibiotics and for its ability to form resilient biofilms (Ciofu and Tolker-Nielsen, 2019; Letizia et al., 2025; Moradali et al., 2017). It is involved in both acute and chronic infections, especially in cases of lung, urinary tract, or cutaneous infections (Mulcahy et al., 2014). For example, *P. aeruginosa* causes lung infections in patients suffering from chronic obstructive pulmonary disease and cystic fibrosis, worsening lung function and reducing life expectancy. In these patients, *P. aeruginosa* forms biofilms in the airways (Kolpen et al., 2022), limiting antibiotic efficacy and making treatment challenging. *P. aeruginosa* biofilms are also involved in cutaneous infections, notably in patients with burn wounds and diabetic wounds (Mulcahy et al., 2014).

Antimicrobial peptides (AMPs), also known as host defense peptides, are found in a large variety of life forms from microorganisms to humans (Hancock and Scott, 2000). These short and generally positively charged peptides are, for some of them, capable of inhibiting or killing different types of Gram-positive or -negative bacteria most commonly by disrupting microbial membranes, or by modulating host defense systems (Hancock and Scott, 2000). Scorpion venom is a rich source of biomolecules with potential therapeutic action, since a transcriptomic study revealed that the venom of the Brazilian scorpion *Tityus stigmurus* presented more than 25% of AMPs transcripts (Almeida et al., 2012). AMPs from scorpion venoms reveal physicochemical and structural characteristics that favor their action in inhibiting different microorganisms, such as bacteria (Furtado et al., 2020; Uzair et al., 2018; Xia et al., 2024). Their helical structure, in conjunction with their cationic charges are important factors that contribute to their mechanism of action, considering that their positive net charge and hydrophobic amino acids may interact with the negatively charged bacterial surface. Thus, these peptides may insert in the membrane generating its destabilization or rupture (Amorim-Carmo et al., 2022; Xia et al., 2024). Specifically, Stigmurin (Stig), a peptide found in the venom of *T. stigmurus,* that shows a positive net charge (+2), is amphipathic, and presents 17 amino acid residues with *C*-terminal amidation (Phe-Phe-Ser-Leu-Ile-Pro-Ser-Leu-Val-Gly-Gly-Leu-Ile-Ser-Ala-Phe-Lys-NH_2_) (Almeida et al., 2012; De Melo et al., 2015). Stig shows an *α*-helix structure (Ser7 to Phe16) with random structure in its *N*-terminal region when studied by NMR spectroscopy in TFE-*d*_2_:H_2_O (40:60, v:v). This peptide was shown to be active against *Staphylococcus aureus in vitro* and in a polymicrobial sepsis model *in vivo* (Daniele-Silva et al., 2016; De Melo et al., 2015). Intending to enhance and expand the spectrum of action of Stig, alterations on its primary sequence were promoted, substituting specific amino acid residues for Lysine, which gives a higher positive net charge to the designed peptides. Indeed, *in vitro* studies showed that four Stig analog peptides, named StigA6 (+4), StigA16 (+5), StigA25 (+5), and StigA31 (+7), displayed a broad-spectrum antibacterial action against both Gram-positive and Gram-negative bacteria with minimum inhibitory concentrations (MICs) in the range of 1.2–18.75 μM (Amorim-Carmo et al., 2019; Parente et al., 2018). Additionally, StigA8 (+4) and StigA18 (+5), demonstrated growth inhibition activity against *S. aureus* clinical isolates (MIC, 1.87–3.75 μM), and antibiofilm activity against both early (4 h) and mature (24 h) biofilm (3.75–7.5 μM) (Furtado et al., 2022). These analog peptides, in the same way as the native peptide, were analyzed by circular dichroism and demonstrated the ability to change conformation depending on the environment. These peptides showed a helix structure in hydrophobic solvents, more pronounced than Stig, and a random structure in aqueous media. This conformational flexibility may be advantageous for their interaction with the bacterial membranes.

Beyond growth inhibition and bacterial killing, some AMPs were shown to affect other phenotypes, as virulence, metabolism, biofilm formation and/or eradication, at sub-inhibitory concentrations (Castillo-Juárez et al., 2022). In this context, we found that Stig and its analogs StigA31 can disperse a preformed biofilm of *P. aeruginosa* in several models of biofilm dispersion at sublethal concentrations. Both compounds led to a reduction in the bacterial biovolume and the exopolysaccharide content of the biofilm matrix in dynamic flow conditions. We further show that Stig, but not StigA31 led to increase membrane fluidity. NMR-based structural characterization of the secondary structure of StigA31 shows that this peptide chain consists of a stable amphipathic *α*-helix flanked by flexible N- and C-terminal extremities, suggesting StigA31 could interact with matrix components and/or bacterial membrane.

## Materials and methods

### Synthesis of stigmurin and its analogs

Stig and its derived analogs (StigA6, StigA8, StigA16, StigA18, StigA25, and StigA31) were previously synthesized using the solid-phase strategy based on the 9-fluorenylmethoxycarbonyl (Fmoc) chemistry using a RINK amide resin with a loading capacity of 40 mmol·g^−1^ (Chan and White, 2000). Correct synthesis and purification were confirmed using mass spectrometry (LCQ Fleet Ion trap mass spectrometer, Thermo Fisher Scientific, USA) and high-performance liquid chromatography (HPLC) (Waters e2695 system equipped with a Waters 2,998 PDA detector, Waters Corporation, USA).

### *In silico* analysis of physicochemical properties

The hydrophobicity, surface charge, and hydrophobic moment of the analog peptides were predicted by the Heliquest web server^1^ (Gautier et al., 2008). The theoretical secondary structure was obtained using the PORTER server^2^ (Pollastri and McLysaght, 2005).

### StigA31 tridimensional structure determination

The three-dimensional structure of the StigA31 was evaluated using the two-dimensional liquid-state nuclear magnetic resonance (NMR) spectroscopy. StigA31 (5 mM) was prepared in a deuterated 2,2,2-trifluoroethanol aqueous solution (40:60%, v/v). The 1H mononuclear total correlation spectroscopy (TOCSY), 1H nuclear Overhauser spectroscopy (NOESY), and 1H-13C heteronuclear single-quantum correlation (HSQC) and 1H-15 N multiple quantum heteronuclear correlation (HMQC) experiments were acquired on an AVANCE DRX 800 nuclear magnetic resonance spectrometer (Brucker^©^). A 5 mm triple resonance probe equipped with a coil was used to employ field gradient pulses at 298 K. TOCSY MLEV-17 pulse sequence (Bax and Davis, 1985) was acquired with a mixing time of 60 ms, rotation lock time of 80 ms and spectral width of 9,615 Hz, with 640 increments in t1 and 32 transients, and 4,096 acquisition points. NOESY spectrum was obtained with a mixing time of 250 ms with 512 increments in t1 and 16 transients and 4,096 acquisition points in each free induction decay (Kumar et al., 1980). HSQC spectrum was acquired showing CH and CH3 correlations in the positive phase and CH2 in the negative phase (Willker et al., 1993). In the HQMC analysis, the SOFAST pulse sequence was used with spectral widths of F1 and F2 of 2,433 Hz and 9,615 Hz, respectively, with 128 increments collected at t1 with 1,024 transients from 1,024 acquisition points (Schanda et al., 2005). The obtained spectra were processed using the NMRPipe software (NMRPipe Spectral Processing and Analysis System) and then analyzed and signals assigned using the NMR View software (Johnson and Blevins, 1994). The reference to identify spin systems was obtained from table of chemical shifts from the Biological Magnetic Resonance Data Bank (available at: https://bmrb.io/ref_info/). Intensities of Nuclear Overhouser Effect (NOE) correlations obtained from the NOESY spectrum were converted into strong (2.8 Å), medium (3.4 Å), and weak (5.0 Å) distance constraints (Hyberts et al., 1992). Chemical shifts were converted into dihedral angles using the TALOS+ software. Three-dimensional structures were calculated using the Xplor-NHI software where 30,000 simulation steps were considered at 1000 K, with a temperature decrease at 9000 steps. The stereochemical quality of the structures was evaluated using PROCHECK-NMR and the analysis of the obtained structures was performed with the Pymol program.

### Strain and growth conditions

*Pseudomonas aeruginosa* H103, a prototroph of the wild-type PAO1 strain obtained from the laboratory of R.E.W. Hancock (University of British Columbia, Vancouver, Canada; Chandler et al., 2019), was cultured in Luria-Bertani broth (10 g. L^−1^) (LB, Difco, BD). The bacteria were grown overnight (18 h) at 37 °C with shaking at 180 rpm. A bacterial suspension was then prepared to an optical density of A_580_ = 0.1 and incubated in LB at 37 °C for 24 h, either with or without shaking at 180 rpm. To evaluate the impact of the peptides on the growth kinetics of *P. aeruginosa* H103, bacterial cultures were incubated with or without the compounds at concentrations ranging from 0.001 to 10 μM. Absorbance at 580 nm (A_580nm_) was measured every 30 min using the Spark 20 M multimode microplate reader, operated with Spark Control™ software (version 2.1, Tecan Group Ltd., Crailsheim, Germany). The resulting data were plotted, with each data point representing the mean ± standard error of the mean (SEM) for the A_580nm_ readings. Each experiment was repeated at least three times.

### MICs evaluation

*P. aeruginosa* H103 was grown in LB overnight, and adjusted to 5.10^5^ CFU.mL^−1^ in LB according to EUCAST recommendations with minor modifications, before being exposed to Stig or its analogs at concentrations ranging between 1 and 100 μM. Growth was performed at 37 °C for 24 h without shaking, and the minimal inhibitory concentration (MIC) was defined for each compound from at least triplicate observations as the lowest concentration of the compound allowing no visible growth. Bacterial viability was assessed by adding resazurin (Acros-organics) at a final concentration of 0.0125 mg.mL^−1^ to the microplate wells. After incubation for 2 h at 37 °C, the color of the wells was observed. A pink or purple color change reflected active cell metabolism, whereas an unchanged color (blue) reflected the MIC.

### Pyocyanin measurement

*P. aeruginosa* H103, either untreated or treated with Stig or its analogs at 0.1 μM, was cultured in LB broth in a 96-well microtiter plate (Thermo Fisher Scientific, Nunc™, Waltham, MA, USA) at 37 °C for 24 h with shaking at 180 rpm. Following the incubation period, bacterial growth was evaluated by measuring the absorbance at 580 nm. Pyocyanin quantification was performed according to the methods described by Azuama et al. (2020) and Tahrioui et al. (2020). Briefly, an equal volume of chloroform was added to the culture supernatant, followed by centrifugation. The blue chloroform phase was collected, and half the volume of 0.5 M HCl was added. After a second centrifugation, the resulting red-pink HCl phase was collected, and the absorbance at 520 nm (A_520nm_) was recorded using the Spark 20 M multimode microplate reader (Tecan Group Ltd., Crailsheim, Germany). The results were normalized to bacterial cell density (A_520nm_/A_580nm_) and expressed as a percentage of the control. Each experiment was repeated four times.

### Fluorescence anisotropy measurement

The membrane fluidity of *P. aeruginosa* H103 cultures untreated and treated with Stig or analogs at 0.1 μM was evaluated using fluorescence anisotropy, as previously described by David et al. (2024). Briefly, the bacteria (A_580nm_ = 0.1) were cultured for 24 h with ultrapure Milli-Q^®^ water (control) or Stig or StigA31 (final concentration of 0.1 μM) in erlenmeyer flasks, with shaking at 180 rpm. Then, the bacterial cells were then centrifuged at 7500 g for 5 min at room temperature. The pellets were washed twice with 10 mM MgSO_4_.7H_2_O and resuspended in the same solution to reach an absorbance at 580 nm (A_580nm_) of 0.1. Next, 1 μL of 4 mM 1,6-diphenyl-1,3,5-hexatriene (DPH) dissolved in tetrahydrofuran was added to 1 mL of the bacterial suspension. The mixture was then incubated at 37 °C for 30 min in the dark to allow the probe to incorporate into the bacterial membranes. Fluorescence polarization was then measured using a Spark 20 M multimode microplate reader (Tecan Group Ltd., Crailsheim, Germany), equipped with an active temperature control system. The excitation and emission wavelengths were set to 365 nm and 425 nm, respectively. Each experiment was repeated three times.

### Biofilm prevention assays

Biofilm formation was assessed using 96-well microtiter plates (Nunc™, Thermo Fisher Scientific, Waltham, MA, USA). Following an overnight growth period, the *P. aeruginosa* H103 culture was diluted to an A_580_ value of 0.1 in LB broth. Stig or its analogs were then added to the inoculum at various concentrations (0.01–10 μM). The biofilms were then incubated at 37 °C under static conditions for 24 h, and the A_580nm_ was recorded. To quantify the biofilm, the medium was discarded, and the wells were rinsed with distilled water. The bound bacteria were then stained with 0.1% crystal violet for 15 min. The crystal violet stain was then dissolved in 30% (v/v) acetic acid, and the absorbance at 595 nm (A_595nm_) was measured using a Spark 20 M multimode microplate reader controlled by Spark Control™ software (version 2.1, Tecan Group Ltd., Crailsheim, Germany). Background staining was corrected by subtracting the values of the uninoculated control wells. The data were normalized to bacterial density (A_595nm_/A_580nm_) and expressed as a percentage of the control.

### Biofilm eradication assays

To eradicate the biofilm, the *P. aeruginosa* H103 overnight culture was adjusted to an A_580nm_ value of 0.1 in LB.

#### Calgary-like devices

Biofilms were grown under static conditions at 37 °C on polystyrene pegs attached to the lids of microtiter plates (Nunc™ Immuno TSP Lids). The pegs were then rinsed with a physiological saline buffer (0.09% NaCl) and treated with Stig or its analogs at various concentrations, or ultrapure Milli-Q^®^ water (control) for 24 h at 37 °C under static conditions. After treatment, the pegs were rinsed three times with distilled water and stained with 0.1% crystal violet for 15 min at room temperature. The crystal violet stain was then solubilized in 30% (v/v) acetic acid, and absorbance at A_595nm_ was recorded using the Spark 20 M multimode microplate reader. Data were presented as a percentage of the control. Data are the results of three independent biological experiments.

#### Static conditions

An overnight culture of *P. aeruginosa* H103 was diluted in LB medium to an optical density of A_580nm_ = 0.1 and 1 mL was transferred to each well of a 24-well glass-bottom plate. Plates were incubated under static conditions at 37 °C for 24 h to allow biofilm formation. After incubation, planktonic cells were gently removed, and the formed biofilms were treated for 2 h or 24 h with 0.1 μM of either Stig, StigA31, or ultrapure Milli-Q^®^ water (control). Following treatment, residual biofilms were washed twice with physiological water (0.09% NaCl) and stained for visualization by confocal microscopy.

#### Dynamic conditions

The flow cell system, which allows continuous bacterial biofilm formation, was assembled, prepared, and sterilized as described by Tolker-Nielsen and Sternberg (2014). Bacterial cells from an overnight culture were adjusted at an A_580nm_ of 0.1, before 300 μL were inoculated into each channel of the flow cell (1 mm × 4 mm x 40 mm Bio centrum, DTU, Denmark). Bacterial adhesion was performed without any flow for 2 h at 37 °C. After 2 h of adhesion, LB medium was pumped with a flow rate of 2.7 mL.h^−1^ at 37 °C for 22 h. Then, the biofilm cells were treated with ultrapure Milli-Q^®^ water (control), or with either Stig or StigA31 (final concentration of 0.1 μM). After 2 h of treatment, the biofilms were labeled, rinsed for 15 min, and visualized by confocal microscopy.

### Confocal laser scanning microscopy (CLSM) and biofilm quantification

For general biofilm structure imaging, SYTO 9 (green) or SYTO 40 (blue) fluorescent nucleic acid stain (5 μM; Invitrogen, Thermo Fisher Scientific) was used, with excitation at 488 nm and 405 nm and emission collected between 500–550 nm and 410-480 nm, respectively. For matrix components labelling, WFA/WFL-FITC (100 μg.mL^−1^; GlycoMatriX), HHA-TRITC (100 μg.mL^−1^; USBiological Life Sciences) were used, with excitation at 488 nm and 543 nm, respectively, and emission collected between 500–550 nm and 560–600 nm, respectively. CLSM imaging was performed using either a STELLARIS confocal microscope (Leica Microsystems, Nanterre, France) or a Zeiss LSM710 system (Carl Zeiss Microscopy, Oberkochen, Germany), both equipped with a 40 × oil immersion objective. Z-stack images were acquired at 1 μm intervals throughout the full depth of each biofilm. Three-dimensional reconstructions and image processing were carried out using Zen 2.1 SP1 software (Carl Zeiss Microscopy, Oberkochen, Germany^3^). Biofilm structural parameters, including average and maximum thickness (μm), and biovolume (μm^3^/μm^2^), were quantified using the COMSTAT2 software^4^ (Heydorn et al., 2000). For each condition, at least three image stacks were analysed from each the independent experiments.

### Statistical analyses

All experiments were carried out at least three times independently and the mean with standard error of the mean (SEM) was calculated and plotted. Statistical significance was evaluated using Prism GraphPad software version 10.4.1. The data were statistically analysed using either ordinary one-way analysis of variance (ANOVA) followed by Tukey’s multiple comparison test or Kruskal-Wallis test followed by Dunn’s multiple-comparison test to calculate *p* values. Significance was considered at ****, *p* < 0.0001; ***, *p* = 0.0001–0.001; **, *p* = 0.001–0.01; *, *p* = 0.01–0.05; ns (not significant), *p* > 0.05.

### NMR-based structural characterization of the secondary structure of StigA31

The three-dimensional structure of StigA31 was determined by two-dimensional liquid-state Nuclear Magnetic Resonance (NMR) spectroscopy at 298 K using a 5 mM sample in deuterated 2,2,2-trifluoroethanol/water mixture (TFE-d_2_:H_2_O, 40:60, v/v). Spectra were acquired on a Bruker AVANCE 800 MHz spectrometer (National Center of NMR Jiri Jonas, University of Rio de Janeiro, Brazil), equipped with 5 mm TXI triple-resonance probes (^1^H/^13^C/^15^N) and z-gradient capability. Water suppression was performed using pre-saturation, with DSS serving as the internal chemical shift reference. The NMR included ^1^H-^1^H Total Correlation Spectroscopy (TOCSY), ^1^H-^1^H Nuclear Overhauser Spectroscopy (NOESY), and ^1^H-^13^C Heteronuclear Single-Quantum Correlation (HSQC). TOCSY spectra were acquired using the MLEV-17 pulse sequence with mixing time of 60 ms, a spin-lock duration of 80 ms, spectral width of 9,615 Hz, 640 t1 increments, 32 transients, and 4,096 acquisition points (Bax and Davis, 1985). NOESY spectrum was obtained with a mixing time of 120 and 250 ms with 512 increments in t1 and 16 transients and 4,096 acquisition points in each Free Induction Decay (FID) (Kumar et al., 1980) HSQC spectra were recorded in edited mode with CH and CH_3_ correlations in the positive phase, CH_2_ in the negative phase (Willker et al., 1993). Spectra were processed with NMRPipe (Schanda et al., 2005) and peptide assignments were completed in CcpNmr Analysis 2.5.2 (Johnson and Blevins, 1994). NOESY-derived peak lists were converted into distance restraints, while dihedral angles (*φ* and *ψ*) were calculated using DANGLE 1.1 and evaluated against allowed Ramachandran regions (Hyberts et al., 1992; Qin et al., 2022). Structural models were generated in CNS Solve 1.21/ARIA 2.3.2 GUI 1.4 using the restraints and angles from CcpNmr and DANGLE (Ben Hur et al., 2022; Pang et al., 2019; Rangel et al., 2020). Twenty initial structures were refined through nine iterative cycles in vacuum, followed by water minimization of the 40 best-scoring conformers, yielding a final ensemble of 10 lowest-energy structures. All models were rendered in PyMOL 2.4.0a0 for visualization (Schrödinger, LLC. The PyMOL Molecular Graphics System, Version 2.4.0a0).

## Results

### Stig and Stig-analogs synthesis, and physicochemical characteristics

Stig, StigA6, StigA8, StigA16, StigA18, StigA25, and StigA31, were synthesized with C-terminal amidation, purity > 95%, and experimental mass equivalent to the theoretical molecular mass. Their physicochemical characteristics are summarized in Table 1.

**Table 1:** Physicochemical characteristics of Stig and analog peptides.

Peptide	Sequence	Net charge	Isoelectric point	Hydrophobicity	Hydrophobic moment	Molecular mass (Kda)
Stig	FFSLIPSLVGGLISAF**K-**NH_2_	+2	9.07	0.89	0.57	1795.20
StigA6	FFSLIP**K**LV**K**GLISAF**K**-NH_2_	+4	9.54	0.78	0.67	1908.16
StigA8	FFSLIP**K**LVG**K**LISAF**K**-NH_2_	+4	9.54	0.78	0.65	1907.42
StigA16	FF**K**LIP**K**LV**K**GLISAF**K**-NH_2_	+5	9.66	0.72	0.72	1949.36
StigA18	FFSLIP**K**LVG**K**LI**K**AF**K**-NH_2_	+5	9.66	0.72	0.70	1948.51
StigA25	FFSLIPSLV**KK**LI**K**AF**K**-NH_2_	+5	9.66	0.72	0.70	1978.53
StigA31	FF**K**LIP**K**LV**KK**LI**K**AF**K**-NH_2_	+7	9.84	0.61	0.80	2060.73

In the peptide sequence, the letters in bold are the amino acid substitutions for lysine (K).

### Stig and Stig-analogs do not affect *P. aeruginosa* growth and biofilm formation

**Figure 1:**
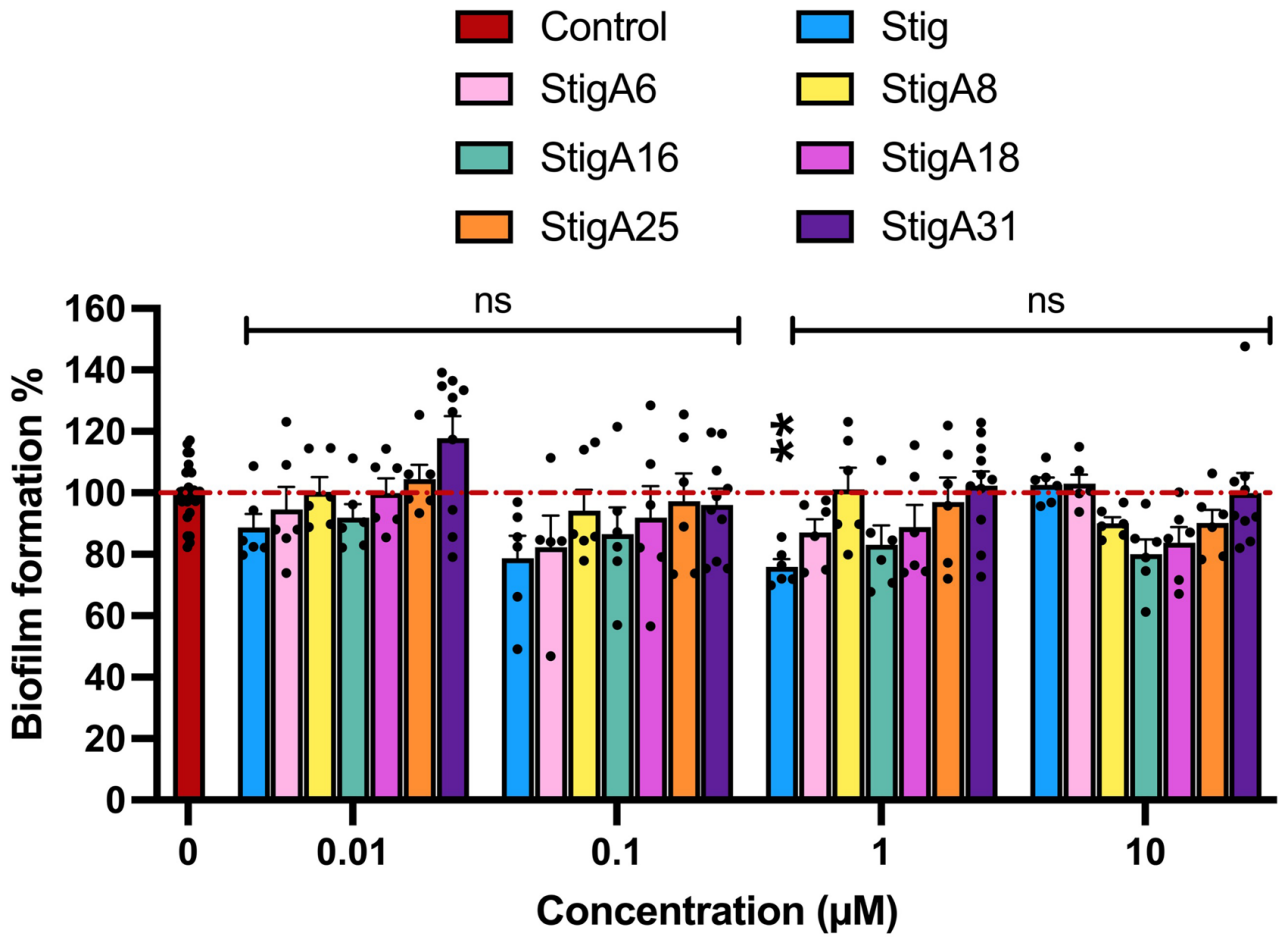
Stig can slightly inhibit biofilm formation. Biofilms were grown for 24 h in microtiter plates with Stig (blue bar), Stig-analogs at concentrations ranging from 0.01 to 10 μM, or water as a control. After crystal violet staining, biofilm formation inhibition was evaluated by colorimetry at 595 nm. The red dotted line corresponds to *P. aeruginosa* H103 biofilm with ultrapure Milli-Q^®^ water as a treatment (control condition). The error bars represent the standard error of the means (SEMs) and are the result of the analysis of three independent biological assays. Statistics were achieved by Kruskal-Wallis test followed by Dunn’s multiple-comparison test. Significance was considered at **, *p* = 0.001–0.01; ns (not significant), *p* > 0.05.

Stig and some of its analogs were previously exhibited antibacterial activity, with MIC values ranging from 1.17 to 9.38 μM against Gram-positive bacteria (*S. aureus* ATCC 29213, *S. epidermidis* ATCC 12228, *Enterococcus faecalis* ATCC 4028). While Stig showed no detectable activity against Gram-negative strains, its analogs displayed Mics ranging from 1.17 to 18.75 μM, notably against *P. aeruginosa* ATCC 27853 (Amorim-Carmo et al., 2019; Daniele-Silva et al., 2016; De Melo et al., 2015; Furtado et al., 2022; Parente et al., 2018). MIC assays were performed on *P. aeruginosa* H103 for each peptide at concentrations ranging from 1 μM to 100 μM in LB medium. In these conditions, no growth inhibition was observed, as confirmed when using resazurin as a metabolic colorimetric control (Supplementary Figure S1). Accordingly, the growth of *P. aeruginosa* H103 treated with Stig, its analogs or water was not altered at concentrations ranging from 1 nM to 100 μM (Supplementary Figure S2). A slight growth slowdown was observed in the case of treatment with StigA18, StigA25, and StigA31 at 100 μM, but a similar final OD was reached (Supplementary Figure S2). We next investigated the peptides’ effects at concentrations ranging from 10 nM to 10 μM on biofilm formation inhibition. As shown in Figure 1, no significant impact was detected for StigA6, StigA8, StigA16, StigA18, or StigA25. Stig led to a reduction in the biofilm formation by 24% at 1 μM, and no significant effect was observed at either higher or lower concentrations. Altogether, these data suggest that Stig and its analogs do not strongly affect *P. aeruginosa* H103 growth and biofilm formation at the concentrations tested.

### Stig and its analogs StigA8, StigA16, StigA18, and StigA31 can disrupt *P. aeruginosa* biofilm grown on polystyrene pegs

**Figure 2:**
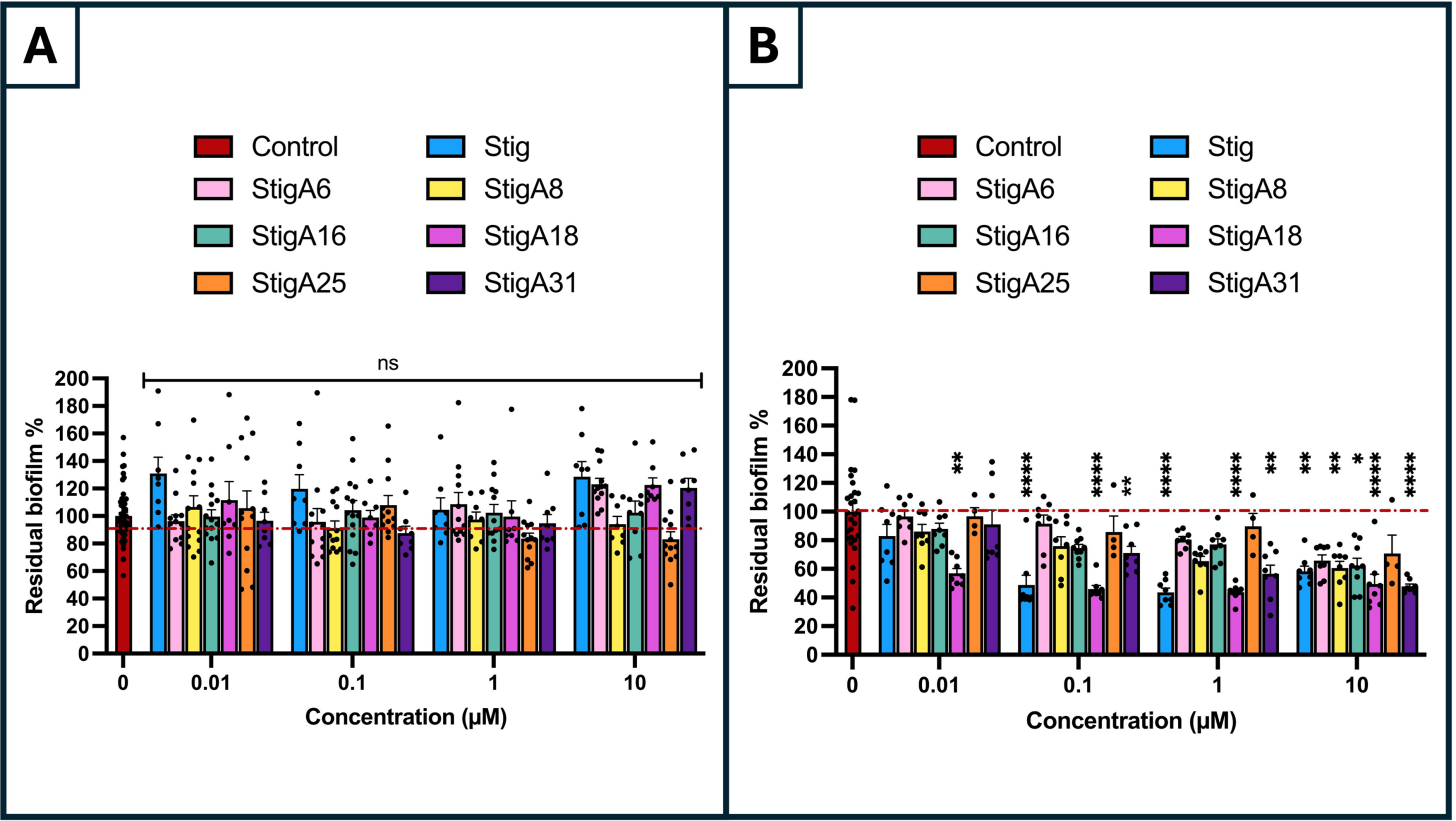
Effect of Stig and Stig-analogs on biofilm disruption upon **(A)** 2 h or **(B)** 24 h treatment at different concentrations in Calgary-like devices. The red dotted line corresponds to *P. aeruginosa* H103 biofilm with ultrapure Milli-Q^®^ water as a treatment (control condition). The error bars represent the standard error of the means (SEMs) and are the result of the analysis of at least three independent biological assays. Statistics were achieved by Kruskal-Wallis test followed by Dunn’s multiple-comparison test. Significance was considered at ****, *p* < 0.0001; ***, *p* = 0.0001–0.001; **, *p* = 0.001–0.01; *, *p* = 0.01–0.05; ns (not significant), *p* > 0.05.

Since biofilms are a major concern in terms of chronic infections, the capacity of these peptides to eradicate pre-established biofilms was assayed at concentrations ranging from 10 nM to 10 μM. To this end, *P. aeruginosa* biofilm was grown on polystyrene pegs for 24 h, then exposed to various concentrations of Stig, its analogs, or water (as a control), for 2 h or 24 h, before crystal violet staining and colorimetric quantification were performed as described in the Materials and Methods section. As shown in Figure 2A, no biofilm dispersion was observed regardless of the peptide considered upon 2 h exposure. By contrast, after 24 h of treatment, Stig was able to disrupt a pre-established 24-h biofilm by approximately 47% at concentrations of 1 and 0.1 μM, and by 32% at 10 μM (Figure 2B). StigA6 and StigA25 showed no significant activity in dispersing biofilms, and StigA8 and StigA16 eradicated 39 and 38% of a pre-established *P. aeruginosa* biofilm at 10 μM. Among the tested peptides, StigA31 and StigA18 showed the best dispersal activity, with quite similar effects. StigA31 exhibited a clear dose-dependent response, leading to 52, 43, and 35% biofilm dispersion at 10, 1, and 0.1 μM, respectively, while StigA18 induced 51, 56, and 55% dispersion at the same concentrations. Overall, these results show that exposure for 24 h to Stig and some of its analogs can disperse a pre-established *P. aeruginosa* biofilm. In the subsequent assays, we chose to restrict our study to the natural peptide Stig, and its analog StigA31 at 0.1 μM.

### Stig and StigA31 effects on the dispersion of *P. aeruginosa* biofilm grown on glass slides in static conditions

**Figure 3:**
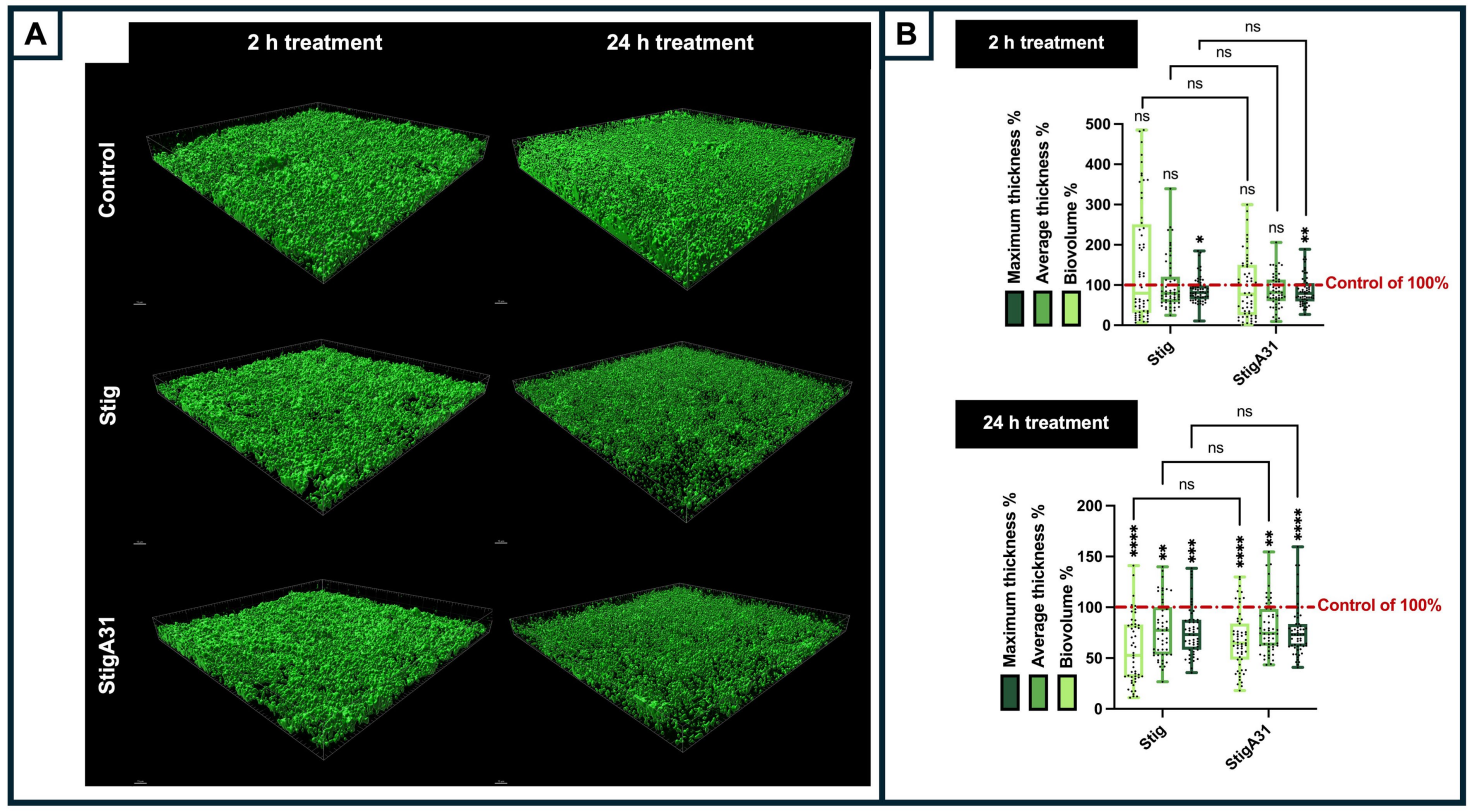
Dispersion of *P. aeruginosa* biofilm driven by Stig and StigA31 under static conditions. **(A)** 3D shadow CLSM images of *P. aeruginosa* H103 biofilms labeled with SYTO9 before (control) and after treatment for 2 h or 24 h with Stig or its analog StigA31 (0.1 μM) under static conditions. Images are representative of six independent biofilm assays (scale bar: 15 μM). **(B)** Quantitative analysis of biofilm was performed using COMSTAT2. Box-and-whisker plots represent the maximum thickness, average thickness, and biovolume expressed as a percentage of the untreated control (set to 100%, red dashed line). Data are the result of the analysis of at least six views of each of the six independent biological assays. Statistical analysis was performed using Kruskal-Wallis test followed by Dunn’s multiple-comparison test. Significance was considered at ∗∗∗∗, *p* < 0.0001; ∗∗∗, *p* = 0.0001–0.001; ∗∗, *p* = 0.001–0.01; ∗, *p* = 0.01–0.05; ns (not significant), *p* > 0.05.

Microtiter plates with polystyrene pegs were used as models to screen for compounds with biofilm-dispersing activity. To confirm this activity in another biofilm culture model, biofilm was allowed to develop for 24 h at 37 °C on 24-well glass-bottom plates. Upon 2 h of treatment with Stig, StigA31, or water (control), and after SYTO-fluorescent labeling, we observed that *P. aeruginosa* H103 displayed a heterogeneous biofilm (Figure 3A). Neither Stig nor StigA31 significantly affected the biovolume, average thickness, or maximal thickness compared to the untreated control (Figures 3A,B). We next investigated whether extending the exposure duration would increase the peptides’ efficacy. Upon 24 h of treatment, the biofilm of *P. aeruginosa* was sparser than upon 2 h exposure. In this condition, treatment with either Stig or StigA31 led to a visible reduction of the biofilm biomass compared to the untreated control, as observed on CLSM images (Figure 3A). Quantitative COMSTAT2 analysis further revealed that Stig reduced the biofilm biovolume, average thickness, and maximum thickness by 42, 21 and 23%, respectively, whereas StigA31 decreased these parameters by 33, 19 and 24% respectively, indicating comparable dispersal effect for both peptides (Figure 3B). Altogether, these data suggest that prolonged exposure is required to observe a significant dispersal effect of Stig and StigA31 in this static biofilm model, with similar outcomes obtained in both microtiter plate and glass-slide models.

### Stig and StigA31 disperse *P. aeruginosa* biofilm grown in a dynamic flow cell device

**Figure 4:**
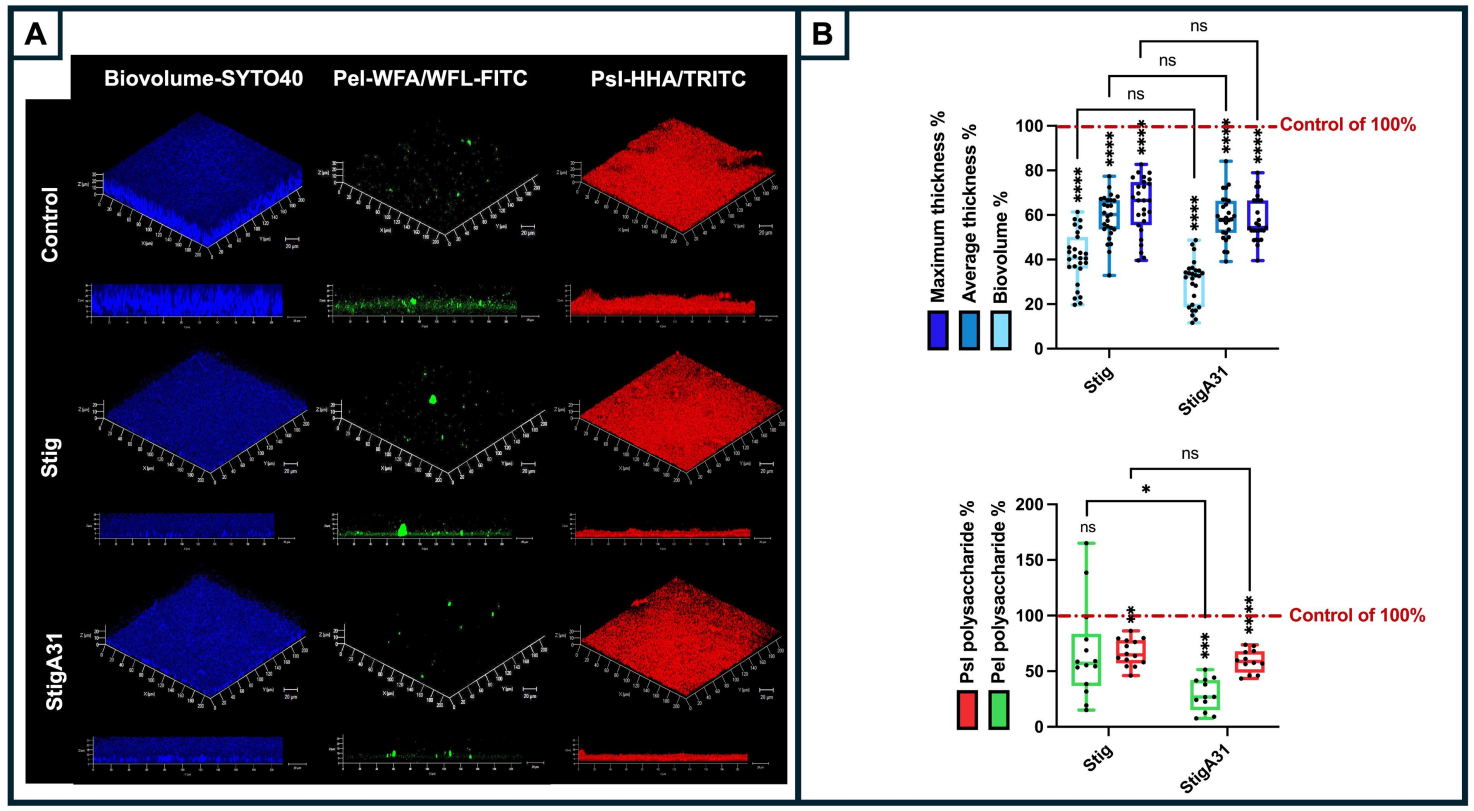
Stig and StigA31 disperse *P. aeruginosa* H103 pre-established biofilm upon 2 h treatment in a dynamic flow cell device and alter its matrix. **(A)** 3D shadow (upper panel), side (middle panel), and top (lower panel) CLSM images representative of a 24 h-old biofilm of *P. aeruginosa* after bacterial labeling with SYTO 40 (blue), Pel polysaccharide with WFA/WFL-FITC (green) and Psl polysaccharide with HHA/TRITC (red) before (control) and upon 2 h of treatment with Stig or StigA31 at 0.1 μM concentration. Images show representative data from five independent biofilm assays (scale bar: 20 μm). **(B)** COMSTAT2 analyses were performed to determine maximum thickness, average thickness and biovolume of the control condition (*P. aeruginosa* H103 treated with ultrapure Milli-Q^®^ water for 2 h), and the assays (*P. aeruginosa* H103 upon Stig or StigA31 treatment for 2 h). The control parameters were set at 100% (red line). The error bars represent the standard error of the means (SEMs) and are the result of the analysis of a minimum of three views of each of the three independent biological assays. Statistics were performed by Kruskal-Wallis test followed by Dunn’s multiple-comparison test. Values that are significantly different are indicated by asterisks as follows: ****, *p* < 0.0001*, p* = 0.001–0.01, *p* = 0.01–0.05; ns, not significant.

We next assayed the effect of a medium flux on the biofilm dispersing activity of Stig and StigA31. To this aim, biofilm was grown for 24 h in a flow cell device with a constant flow of 2.7 mL.h^−1^, after which Stig, StigA31, or water were added for 2 h without flow. After SYTO 40 blue-fluorescent labeling, *P. aeruginosa* H103 displayed a flat, and homogeneous biofilm architecture (Figure 4A). Adding Stig or StigA31 led to strongly dispersed *P. aeruginosa* biofilm (Figure 4A). As shown in Figure 4B, exposure to Stig or StigA31 led to a reduction in the biofilm biovolume by 59 and 70%, respectively. Similarly, both peptides affect the biofilm maximal and average thicknesses, with a reduction of about 40% in each case (Figure 4B). Since the two peptides alter the biofilm architecture, we next investigated their effect on the major exopolysaccharides, by labeling Psl with the pure Hippeastrum hybrid lectin (HHA) from *Amaryllis* conjugated to the red TRITC that is specific to mannose-rich polysaccharides, and Pel with the *Wisteria Floribunda* lectin conjugated to the green fluorescein that is selective for *N-*acetyl-galactosamine residues (Figure 4A). Stig treatment led to a reduction in Psl detection by about 33%, and no significant effect could be observed on Pel (Figures 4A,B). StigA31 exposure results in lower detection of both Pel and Psl within the biofilm matrix (Figure 4A), by 71, and 41%, respectively (Figure 4B). Altogether, these data indicate that StigA31, and to a lower extent Stig, can disperse a pre-established *P. aeruginosa* biofilm, by reducing the biofilm biovolume and maximal and average thicknesses, and by affecting the exopolysaccharide detection within the biofilm matrix.

### Effect of Stig and StigA31 on pyocyanin production

The planktonic lifestyle of pathogenic *P. aeruginosa* is associated with acute virulence via the production of virulence factors. We thus asked if a treatment with these peptides would lead *P. aeruginosa* to produce more virulence factors. To address this question, the production of pyocyanin, one of the many toxic compounds produced and secreted by *P. aeruginosa* was assayed. Pyocyanin is a blue fluorescent phenazine that induces oxidative stress, thereby killing other microorganisms, as well as eucaryotic cells (Gonçalves and Vasconcelos, 2021). *P. aeruginosa* was exposed to Stig, or StigA31 at 0.1 μM for 24 h, before pyocyanin was extracted and quantified as described in the Materials and Methods section. While StigA31 did not result in pyocyanin production alterations, a Stig exposure led to a marginal but significant reduction of the amount of extracellular pyocyanin (about 5%, Supplementary Figure S3). Overall, these data suggest that exposure to these two peptides did not substantially affect the pyocyanin production in our conditions.

### Effect of Stig and StigA31 on *P. aeruginosa* membrane fluidity

**Figure 5:**
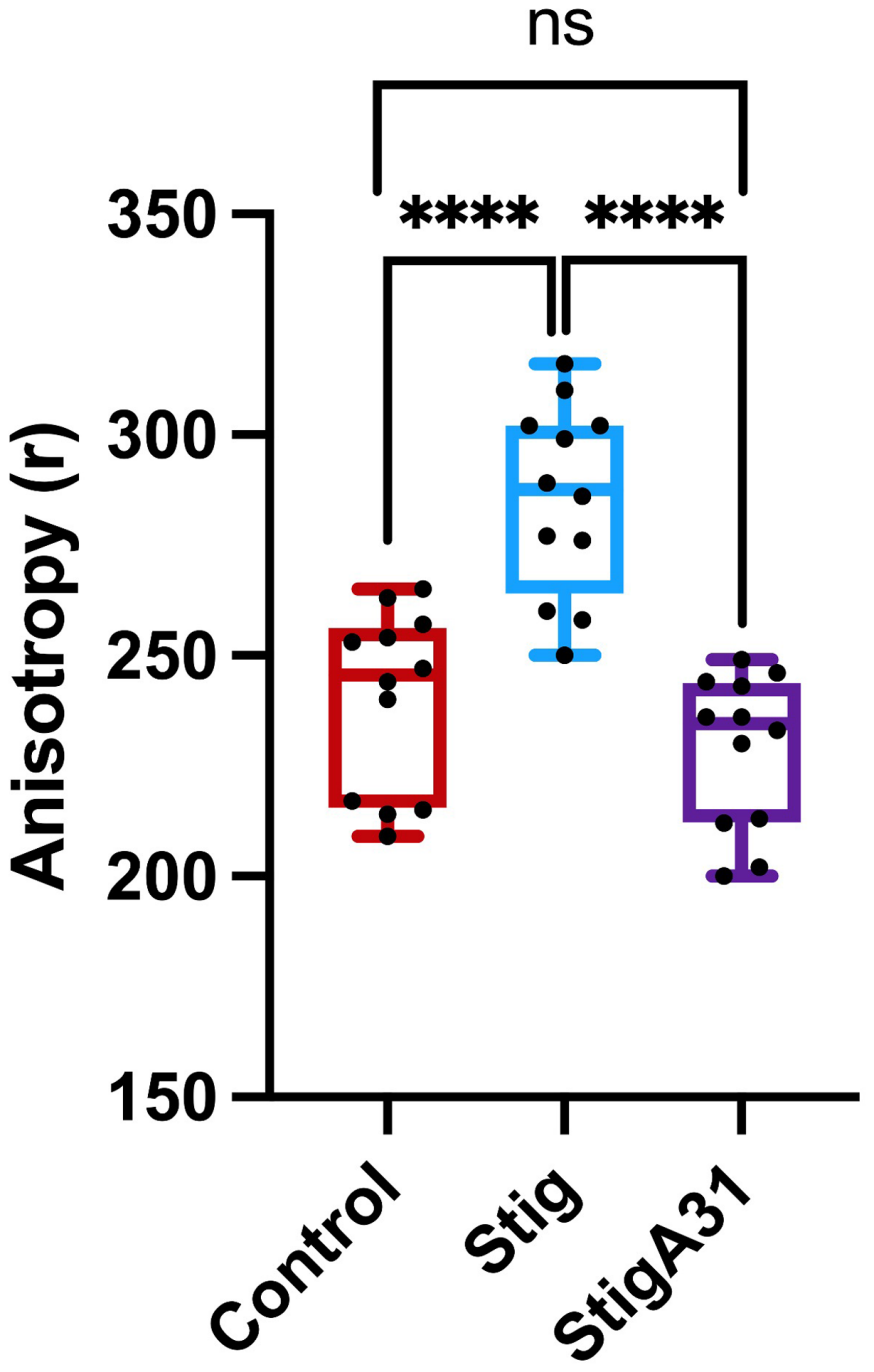
Stig, but not StigA31, increased membrane stiffness. Membrane fluidity assessment by fluorescence anisotropy using 1,6-diphenyl-1,3,5-hexatriene (DPH) probe in *P. aeruginosa* H103 upon treatment with ultrapure Milli-Q^®^ water (red bar), Stig (blue bar), or StigA31 (purple bar). Each experiment was assayed four times independently. Statistics were performed by ordinary one-way ANOVA followed by Tukey’s multiple-comparison test. Significance was considered at ****, *p* < 0.0001; ***ns (not significant), *p* > 0.05.

Since these peptides harbor a distinct number of positive charges (Table 1), we hypothesized that they interact with the negatively charged bacterial membrane, potentially altering the bacterial membrane fluidity. Alterations of membrane fluidity were evaluated by fluorescence anisotropy at the level of the hydrophobic core of the membrane, using the 1,6-diphenyl-1,3,5-hexatriene (DPH) hydrophobic probe. As shown in Figure 5, a fluorescence anisotropy increase was observed upon Stig treatment, suggesting an increased stiffness of *P. aeruginosa* membrane in response to this peptide. No effect was observed in response to StigA31, suggesting that they could act on bacteria through a different mode of action.

### StigA31 folds into an *α*-helix structure in hydrophobic environments

**Figure 6:**
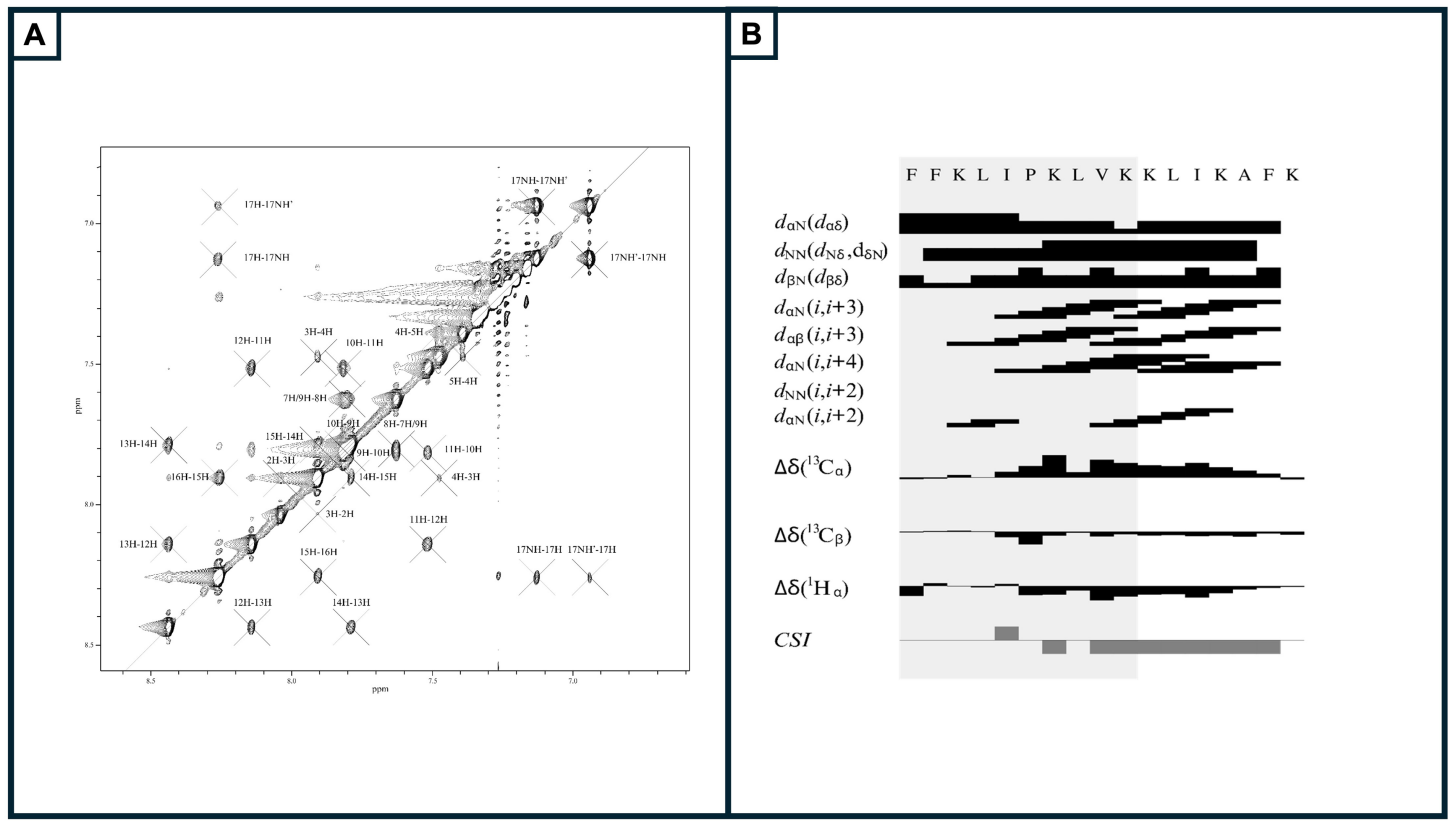
**(A)** Sequential HN-HN (*i*, *i* + 1) correlations observed in the ^1^H-^1^H NOESY spectrum of StigA31 (5 mM) in TFE-d_2_:H_2_O (40:60%, v:v) at 298 K, and **(B)** NOE correlations of StigA31 (5 mM) in TFE-d_2_:H_2_O (40:60%, v:v) at 298 K obtained by bidimensional liquid NMR.

Finally, to get some insights about the possible mode of action of StigA31 on bacterial membranes, NMR spectroscopy was used to characterize the three-dimensional structure of StigA31 in TFE-*d*_2_:H_2_O (40:60, v/v), since similar assays were previously performed on the native Stig peptide (Daniele-Silva et al., 2021). Combined interpretation of the contour maps from the ^1^H TOCSY, ^1^H NOESY, ^1^H-^13^C HSQC, and ^1^H-^15^N HSQC experiments allowed the assignment of the chemical shifts (Supplementary Table S1). NOE nonsequential correlations consistent with *α*-helical organization were identified, including medium- and long-range interactions such as Hα–NH and Hα–Hβ (Supplementary Figure S4). Furthermore, sequential correlations revealed a continuous network of sequential and intra-residue interactions, confirming the primary sequence (Figure 6A, Supplementary Figures S5, S6). NOE correlations converted to distance constraints indicate the presence of an α-helix in the center of the peptide chain, flanked by flexible and disordered termini (Figure 6B). Sequential NOEs are consistently observed across several consecutive residues (d_αδ_; d_Nδ_,d_δN_; and d_βδ_), supporting molecular ordering. The helical coiling is further evidenced by non-sequential NOEs, such as *i*,*i* + 3 and *i*,*i* + 4, detected in the central residues. Complementarily, NOEs between hydrophobic side chains of Phe, Leu, Ile, and Val suggest stable lateral packing, consistent with an amphipathic helix. Chemical shifts reinforce this interpretation: negative Δ^1^H_α_, positive Δ^13^C_α_ and, negative Δ^13^C_β_ are observed systematically, as confirmed by the Chemical Shift Index (CSI), which highlights a well-defined helical conformation in the central core, whereas the termini lack this pattern. Proline-6 locally disrupts sequential NOEs, causing a perturbation, while near of the N- and C-termini weaker signals are observed, typical of increased conformational flexibility.

**Figure 7:**
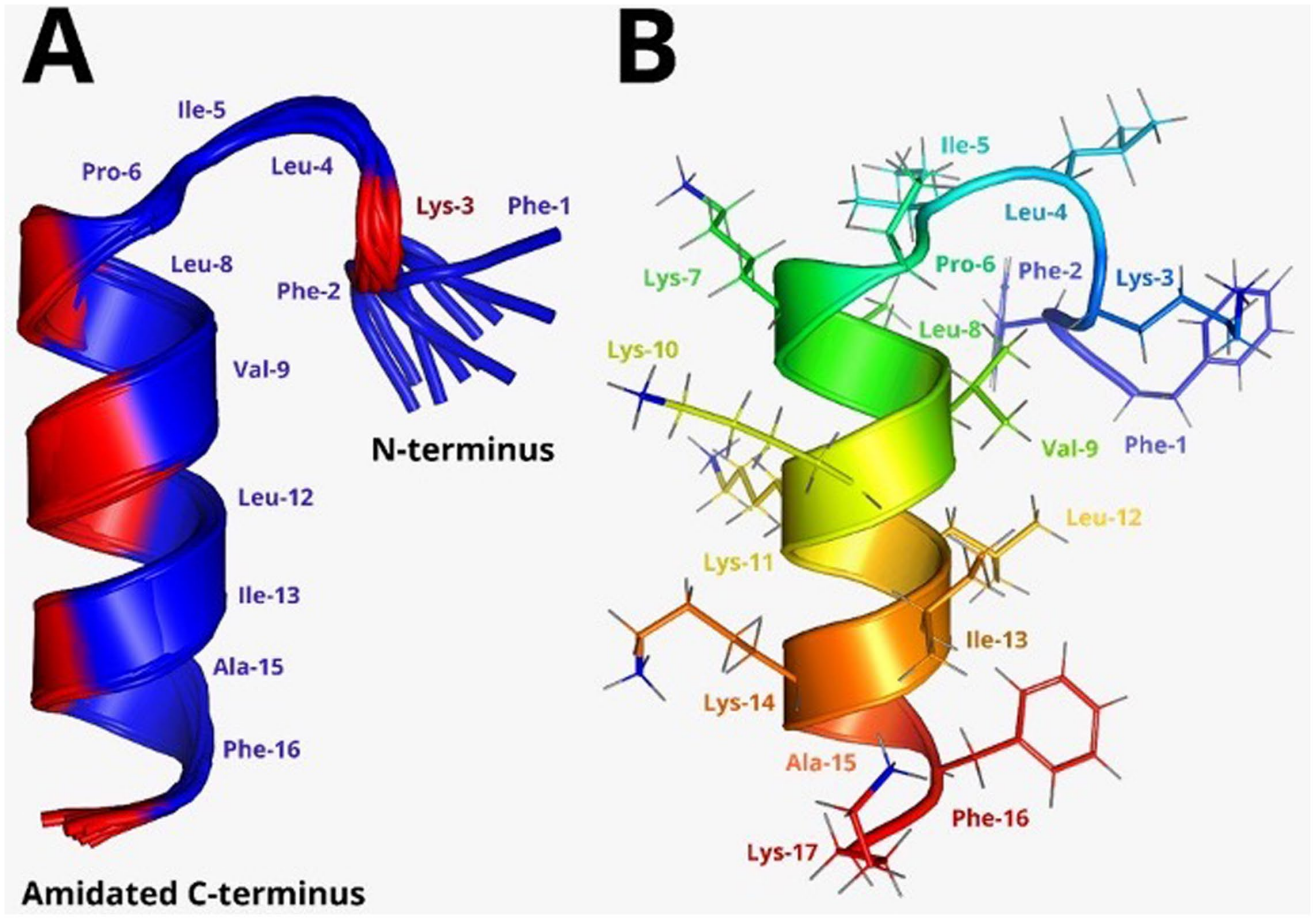
Structures of StigA31 determined by NMR in TFE-d_2_:H_2_O 40:60 (v/v). **(A)** Ten representative structures, colored by residue polarity (hydrophobic in blue, hydrophilic in red) and aligned based on the lowest energy. **(B)** Lowest-energy structure with explicit representation of side chains.

NMR data were employed to predict dihedral angles and assess the overall structural quality of the peptide. Ramachandran analysis revealed that 94% of the residues were located in favored regions and 6% in allowed regions, with none in disallowed areas, demonstrating the absence of geometric distortions and confirming stereochemical reliability (Supplementary Figure S7). The resulting structural models were well defined, supported by numerous unambiguous distance restraints, dihedral angle restraints, and only a few ambiguous restraints. Structural consistency was further validated by the low global RMSD (Root Mean Square Deviation) values, which indicate high convergence, precision, and stability, particularly within the ordered regions of the peptide. A summary of these results is provided in Table 2. The ten most stable structures of the StigA31 (Figure 7A), obtained after final refinement in aqueous solution, were selected to represent its structural model and aligned based on the lowest-energy structure (Figure 7B). Structural analyses indicate that the peptide adopts a stable and amphipathic α-helix spanning residues Pro6 to Phe16, with a helical content of 64.7%. This spatial arrangement of the peptide backbone supports both conformational stability and the potential for selective interactions in biological environments.

**Table 2:** Summary of NMR-derived structural calculations and validation statistics for StigA31 (5 mM) by NMR in TFE-d2:H_2_O (40:60%, v:v) at 298 K.

Experimental restraints	StigA31 data
Unambiguous Distance restraints	408
Intra-residue	236
Sequential (*i*, *i* + 1)	104
Medium range (*i*, *i* + 2)	16
Medium range (*i*, *i* + 3)	42
Large range (*i*, *i* + 4)	10
Ambiguous Distance restraints	60
Dihedral angle restraints	26
Ensemble RMSD (10 structures)	
All residues	
Backbone (N, CA, C, O)	0.64 ± 0.23 Å
Heavy atoms	1.45 ± 0.28 Å
Ordered residues	
Backbone (N, CA, C, O)outra	0.30 ± 0.12 Å
Heavy atoms	0.85 ± 0.18 Å
Ramachandran region statistics (all residue types)	
Core	94.0%
Allowed	6.0%
Disallowed	0.0%

## Discussion

Biofilms play a pivotal and multifaceted role in chronic infections, acting as a sophisticated survival strategy for bacteria that profoundly impacts persistence, immune interactions, antibiotic resistance, and clinical management. They are common in chronic wounds, medical device-related infections, respiratory diseases, and other persistent infections. Thus, it is urgent to find alternative therapeutic strategies and new active molecules, particularly to counter such biofilm-related chronic infections.

In this study, we investigated the activity of Stig, a peptide isolated from the venom gland of the Brazilian scorpion *Tityus stigmurus*, and its derived analogs (StigA6, StigA8, StigA16, StigA18, StigA25 and StigA31) against *P. aeruginosa* biofilms. We show that Stig and its analogs have little effect on *P. aeruginosa* H103 biofilm formation at concentrations ranging from 10 nM to 10 μM. We then investigated their effects on the dispersion of a pre-established biofilm, and showed that exposure to Stig and some of its analogs for 24 h can efficiently disperse a pre-established *P. aeruginosa* biofilm by about 52 and 51% for StigA31 and StigA18, respectively at 10 μM and 43 and 56% at 1 μM, and 35 and 55% respectively, at 0.1 μM. Notably, no growth alterations were observed for any of the tested peptides at concentrations ranging from 10 nM to 10 μM. This result was supported by the absence of MIC in our conditions, indicating that the reduced biofilm biomass detected after treatment was not due to growth inhibition or bacterial killing. Similar data were previously described for the aminosterol squalamine and its derivative claramine A1 under similar technical conditions (Calgary-like devices, and crystal violet staining). These compounds display about 40% dispersion at 1 μM, and 40% (squalamine) and 30% (claramine A1) at 0.1 μM (Tareau et al., 2025). However, while the venom peptides were more efficient after 24 h, the aminosterols display the greatest efficacy upon 2 h exposure (Tareau et al., 2025), suggesting that their modes of action would be different. However, despite no growth alterations were observed in our conditions, it is possible that the extracellular matrix may facilitate peptide retention and local enrichment, thereby increasing their effective concentration within the biofilm environment (Flemming and Wingender, 2010; Schwartz et al., 2020). So, even if the peptide concentration used to disperse a pre-established biofilm was very low (0.1 μM), is it possible that the local peptide concentration would reach concentrations over 100 μM. In addition, the activity of Stig, StigA31 and StigA18, in terms of bacterial dispersion and effective concentrations is consistent with that of the native dispersion cue, *cis*-2-decenoic acid (cis-DA). Indeed, cis-DA at the concentration of 0.31 μM was capable of naturally releasing 33 to 55% of the biofilm population (Amari et al., 2013; Rahmani-Badi et al., 2015). Interestingly, StigA8 and StigA18 were previously shown to disperse a pre-established biofilm of *S. aureus* (ATCC 29213) by approximately 80% at 12.5 μM, this concentration being however above the MIC of the peptides (2.34 μM, Furtado et al., 2022). Altogether, these data suggest that Stig and some of its analogs, especially StigA31 and StigA18, at concentrations of 1 μM or 0.1 μM, are of great interest to disperse *P. aeruginosa* population within the biofilm structure, without affecting its growth. The latter phenotype is of importance, particularly given the increasing prevalence of antibacterial resistance, as it can be assumed that the less the bacteria are exposed to selection pressure, the less their bacterial genome would evolve by mutations and develop resistance (Cabot et al., 2016). We choose to focus on Stig and StigA31 at a final concentration of 0.1 μM in the subsequent assays.

Concerning the MICs, Stig was previously shown to display a bactericidal activity against the Gram-positive *S. aureus* ATCC 29213 and ATCC 33591 methicillin-resistant strains, with MICs of 8.7 and 17.4 μM respectively, but neither on the Gram-negative *Escherichia coli* ATCC 25922, (MIC>139 μM; De Melo et al., 2015), or on *P. aeruginosa* ATCC 27853 (MIC>150 μM, Parente et al., 2018). By contrast, Stig-analogs, including StigA6 and StigA16 (Parente et al., 2018), StigA25 and StigA31 (Amorim-Carmo et al., 2019), and StigA8 and StigA18 (Furtado et al., 2022) displayed MICs against *P. aeruginosa* ATCC 27853 in a concentration range of 1.17 to 9.8 μM (Amorim-Carmo et al., 2019; Parente et al., 2018). However, in our conditions, none of the peptide tested (1–100 μM) inhibited the growth of *P. aeruginosa* H103, and no MIC values could be determined. While the MICs assays were performed in EUCAST conditions in the previously published studies, herein, we evaluated the effect of the peptides in the conditions used to assay their activity on biofilm dispersion (LB medium, no shaking, microtiter plates), on the model *P. aeruginosa* H103, a prototroph derivative of PAO1 strain. For instance, *P. aeruginosa* H103 was grown in microtiter plates in LB medium without shaking for 24 h before the growth inhibition was evaluated. Such differences may account for the discrepancy in terms of the MICs observed.

Returning to the main result of this study, we next asked whether the biofilm disruption activity of Stig and StigA31 at a concentration of 0.1 μM would be as efficient in other biofilm culture models, as it was on polystyrene microtiter plates. Thus, we performed assays on glass slides in static and in dynamic flow conditions, and we show that the peptides’ efficacy was dependent on the biofilm-growth model and the duration of exposure. As previously mentioned, Stig and StigA31 dispersed about 47 and 35%, respectively, of the *P. aeruginosa* population grown on polystyrene pegs after 24 h of treatment, whereas no significant dispersion was observed after 2 h. When bacteria were grown on glass in static conditions, a similar trend was observed: neither peptide induced significant biofilm dispersion after 2 h of treatment, while both Stig and StigA31 significantly reduced biofilm parameters after 24 h exposure. Finally, when biofilms were grown on glass slides in dynamic flow conditions, both peptides at a concentration of 0.1 μM led to a reduction in the biofilms by about 59 and 70%, respectively, upon 2 h exposure. Interestingly, in the same technical conditions, the human Atrial Natriuretic Peptide (hANP) and the hormone osteocrin were found to disperse a pre-established *P. aeruginosa* biofilm by 77% at 0.5 μM (Louis et al., 2022) and by 59% at 0.01 μM (Louis et al., 2023), respectively.

These differences in terms of dispersal activity can be explained with regard to the techniques used and the type of biofilms formed. On polystyrene pegs, *P. aeruginosa* has to swim to reach the polystyrene surface in order to adhere to it, and to develop a biofilm at the solid–liquid interface. Once the bacteria have attached, the pegs are rinsed to eliminate non-adherent bacteria before crystal violet staining. This system is simple and has the advantage of being less affected by cell sedimentation than the common microtiter assay (Han and Lee, 2023). When *P. aeruginosa* is grown on glass slides in static conditions, two types of biofilms can develop: one at the bottom of the wells at the solid–liquid interface, and one at the air-liquid interface (the pellicle). We exposed a 24 h-preestablished biofilm for 2 h or 24 h with peptides or water as a control, meaning that bacteria were allowed to grow for 24 h or 48 h, respectively. In the latter case, both types of biofilms grew, with the possibility of sedimentation of the biofilm at the bottom of the well, as well as dispersed cells, thereby artificially increasing the biomass of the biofilm (Crivello et al., 2023). Nevertheless, despite these structural particularities, both static systems displayed a similar-time dependent dispersal profile, with no significant effect observed at 2 h and a reduction detected after 24 h exposure. Finally, the dynamic flow system enables continuous supply with fresh medium and nutrients, while removing the planktonic and dispersed cells (Azeredo et al., 2017). In our conditions, this system resulted in biofilm dispersion with low data heterogeneity, upon 2 h Stig or StigA31 exposure, suggesting that this system was possibly the most accurate for assaying the peptides’ efficacy in our conditions. Overall, we demonstrate that Stig and StigA31 can efficiently disperse *P. aeruginosa* population regardless of the biofilm growth system used. However, all of these systems allow biofilms to grow on abiotic surfaces such as glass or polystyrene, which are far from the clinical situation, in which bacteria interact on one side, with host cells and with other microorganisms on the other side. Thus, while the *in vitro* anti-biofilm activities of Stig and its analog StigA31 are convincing, the next step will be to validate their dispersal activity on relevant *ex vivo* and *in vivo* cellular models.

Stig and StigA31 were capable of dispersing *P. aeruginosa* cells regardless of the *in vitro* system used to culture the biofilm in our study. The next question, then, was how the peptides disrupted the biofilm. One hypothesis relies on the possible interactions of the peptides with the biofilm matrix components. The extracellular matrix is the main structural component of biofilm, accounting for up to 90% of its total mass (Flemming and Wingender, 2010). It forms a complex three-dimensional network of exopolysaccharides, including the cationic Pel and neutral Psl, along with eDNA, proteins, and lipids, which together provide cohesion, protection, and mechanical stability of biofilm. During the dispersion processes, it is well established that the components of this matrix are profoundly altered, leading to a partial disorganization or degradation of the matrix (Cherny and Sauer, 2020). Stig and StigA31 are small, linear, cationic peptides with a partly *α*-helical structure and flexible N-terminus, characterized by NMR spectroscopy (Amorim-Carmo et al., 2019, this study), which display 2 and 7 positive charges, respectively. It is possible that the peptides would interact with negatively charged matrix components leading to disruption and bacterial release. We observed that exposure to StigA31 led to a reduction in both the exopolysaccharides Pel and Psl labeling, while treatment with Stig affects Psl detection. Interestingly, Pel binds eDNA through ionic interactions, forming the structural core in the stalk of the *P. aeruginosa* biofilm (Jennings et al., 2015). It is possible that StigA31, and to a lesser extent Stig, would compete with or displace Pel to bind eDNA, leading to partial matrix disorganization. Recently, molecular docking analysis and molecular dynamics simulations revealed strong interactions and stability between the two Stig analogs StigA6 or StigA16 and lectin LecA from *P. aeruginosa* (Thakur et al., 2025), suggesting that Stig and its analogs could interact with matrix components. Interestingly, LecA, a carbohydrate-binding lectin, is a Ca^2+^-dependent tetravalent protein crucial for biofilm integrity, which is thought to link bacteria to the biofilm matrix exopolysaccharides (Diggle et al., 2006). In addition, another major lectin of *P. aeruginosa*, LecB was recently demonstrated to bind to Psl, leading to increased retention of both cells and exopolysaccharides in a growing biofilm, and to coordinate Psl localization within the biofilm (Passos Da Silva et al., 2019). Altogether, these data suggest that these cationic amphipathic peptides could interact with matrix components at least through electrostatic interactions, thus disturbing the matrix and possibly releasing bacteria. While our observations suggest that the peptides may affect matrix organization, a quantitative assessment of extracellular polymeric substances (EPS) was not performed in this study. Such analyses would provide complementary insights into the matrix disruption and help to better understand the contribution of matrix-targeting mechanisms in the observed biofilm dispersal.

In addition to potentially altering the composition and/or organization of the matrix, Stig and some of its analogs have been previously suggested to interact with the negatively charged lipids that are exposed on the outer leaflet of bacterial membranes (De Melo et al., 2015). Stigmurin presents a net positive charge of +2. Modifications to its primary sequence, involving the substitution of neutral amino acids with lysine residues, resulted in an increased net positive charge in the designed peptides, ranging from +4 to +7, while maintaining or enhancing the hydrophobic moment and *α*-helical structure in hydrophobic environments. Regarding the antibiofilm activity, the net positive charge, mainly due to the presence of lysine residues, may promote interactions between these peptides and negatively charged components of the matrix, leading to biofilm disruption and bacterial cell release. In addition, these peptides may interact with negatively charged lipids in the outer leaflet of bacterial membranes or with the lipopolysaccharide layer, thereby destabilizing the outer membrane. Furthermore, four analog peptides, including StigA31, were shown in previous *in silico* studies to interact with membrane models mimicking Gram-negative bacteria. These predictions also indicated that the lysine residues introduced into the sequences were a major factor contributing to this interaction (Amorim-Carmo et al., 2019; Furtado et al., 2022). Here we show that exposure to sublethal concentrations of Stig, but not StigA31 decreases *P. aeruginosa* membrane fluidity. Despite its higher positive charge, StigA31 may interact differently with bacterial membranes. Although cationic charge promotes electrostatic attraction to negatively charged bacterial surfaces, an excessive charge may limit peptide insertion into the lipid bilayer, favoring surface association rather than deep membrane perturbation. One possible explanation is a direct interaction between the peptides and the bacterial membrane with Stig displaying two net positive charges, and StigA31, seven. Stig may directly interact with the bacterial membrane, in particular with negatively charged components such as lipopolysaccharides (LPS), leading to changes in membrane organization and fluidity. By contrast, StigA31, due to its higher charge, may remain predominantly associated with the membrane surface without inducing significant perturbation of membrane structure. It can be hypothesized that the moderate cationic charge of Stig may allow interactions with membrane components in a manner analogous to divalent cations such as Ca^2+^. Another possibility is that the observed changes in membrane fluidity may result from indirect effects related to envelope stress responses. Membrane perturbation by compounds is known to trigger stress signaling pathways, such as those involving the ECF sigma factor SigX, which plays a role in membrane homeostasis in *P. aeruginosa.* Stig would induce a cell envelope stress response (Chevalier et al., 2022), mediated by the ECF sigma factor SigX (Chevalier et al., 2019). This ECF sigma factor is involved in *P. aeruginosa* membrane homeostasis (Fléchard et al., 2018). Since its activity triggers the production of small-chain fatty acids, thus increasing membrane fluidity (Blanka et al., 2014; Boechat et al., 2013), it could be possible that SigX displayed a lower activity, thus reducing the membrane fluidity. These results suggest that the decrease in membrane fluidity observed with Stig may arise either from direct membrane interactions or from stress-induced physiological adaptations, and that membrane modulation is not the sole mechanism underlying biofilm dispersal. Further studies will be required to determine the contribution of these mechanisms.

NMR data indicate that the StigA31 peptide chain consists of a stable amphipathic α-helix between residues Pro6-Phe16 and flanked by flexible N- and C-termini. This conformation is supported by sequential long-range NOEs, hydrophobic side-chain packing, and characteristic chemical shift patterns. Ramachandran analysis and low RMSD values confirm the reliability and high structural convergence of the calculated models. StigA31 displays a helical content of 64.7%. Similar data were described for Stig, but with a shorter alpha-helix domain (58.8%, Amorim-Carmo et al., 2019). It is possible that the StigA31 extended helical region could be relevant to its activity profile in terms of biofilm dispersion. Interestingly, previous studies have shown that Stig and StigA31 can modify their structural conformation according to the environment using circular dichroism assays (Amorim-Carmo et al., 2019; De Melo et al., 2015). Both peptide structures were stable in a large range of pH and temperature (Amorim-Carmo et al., 2019; De Melo et al., 2015). The capacity of such cationic peptides without disulfide bridges to modify their structural conformation according to the environment may provide a flexibility that could be important to the interaction with the biofilm matrix, and/or with the bacteria, which could contribute to the dispersal activity.

Finally, when comparing the biocompatibility of Stig and its analogs, the prototype molecule exhibits very low *in vitro* hemolytic activity (below 20% at 150 μM) and does not significantly affect the viability of normal cell lines, such as canine kidney epithelial cells MDCK (ATCC CCL-34) and murine macrophages RAW 264.7 (ATCC TIB-71), up to a concentration of 20 μM (Daniele-Silva et al., 2016). Regarding the designed analogs, they also demonstrated low percentages of hemolysis at the tested concentrations, reaching approximately 20% only at the highest evaluated concentration (150 μM) for StigA8 and StigA18 and 30% (75 μM) for StigA6 and StigA16 (Parente et al., 2018; Furtado et al., 2022). In addition, no signs of *in vivo* toxicity were observed, such as death, reduced motility, or melanization, in Galleria mellonella larvae at a dose of 120 mg/kg for the StigA8 and StigA18 (Furtado et al., 2022). In contrast, StigA25 and StigA31 exhibited higher hemolysis rates at 150 μM, corresponding to 100% and approximately 80%, respectively. However, at concentrations below 9.4 μM, hemolysis progressively decreased to values below 20%, reaching nearly 0% at the lowest concentration tested (1.2 μM) (Amorim-Carmo et al., 2019). Considering that the activity of StigA31 in the present study was observed between 0.1 and 0.01 μM, it can be inferred that this concentration range does not present relevant hemotoxic potential. Therefore, the evaluated peptides demonstrate satisfactory biocompatibility at the concentrations employed in the present study.

To conclude, this study highlights the potential of Stig and StigA31 as *P. aeruginosa* biofilm-dispersing adjuvants of conventional antibiotics that can be used at sub-inhibitory concentrations, offering a promising approach against biofilm-related chronic infections. However, even if limited, the risk that these compounds could allow the dissemination of planktonic bacteria involved in acute infections, even in the presence of antibiotics, cannot be neglected. The ability to disrupt biofilms without significant bactericidal activity may help reduce resistance development. Further studies focusing on the molecular mechanisms involved in biofilm dispersion of Stig and its analogs are needed to better understand their interactions with bacteria and/or matrix components, and to advance these compounds, particularly StigA31, toward clinical applications.

## Data Availability

The original contributions presented in the study are included in the article/Supplementary material, further inquiries can be directed to the corresponding author.

## Footnotes

1
https://heliquest.ipmc.cnrs.fr/index.html

2
http://distilldeep.ucd.ie/porter/

3
https://www.zeiss.com/microscopy/int/downloads/zen.html

4
http://www.imageanalysis.dk/

## References

[ref1] Almeida D. D. Scortecci K. C. Kobashi L. S. Agnez-Lima L. F. Medeiros S. R. B. Silva-Junior A. A. . (2012). Profiling the resting venom gland of the scorpion Tityus stigmurus through a transcriptomic survey. BMC Genomics 13:362. doi: 10.1186/1471-2164-13-362, 22853446 PMC3444934

[ref2] Amari D. T. Marques C. N. H. Davies D. G. (2013). The putative Enoyl-coenzyme a hydratase DspI is required for production of the *Pseudomonas aeruginosa* biofilm dispersion autoinducer cis −2-Decenoic acid. J. Bacteriol. 195, 4600–4610. doi: 10.1128/JB.00707-13, 23935049 PMC3807434

[ref3] Amorim-Carmo B. Daniele-Silva A. Parente A. M. S. Furtado A. A. Carvalho E. Oliveira J. W. F. . (2019). Potent and broad-Spectrum antimicrobial activity of analogs from the scorpion peptide Stigmurin. Int. J. Mol. Sci. 20:623. doi: 10.3390/ijms20030623, 30709056 PMC6387013

[ref4] Amorim-Carmo B. Parente A. M. S. Souza E. S. Silva-Junior A. A. Araújo R. M. Fernandes-Pedrosa M. F. (2022). Antimicrobial peptide analogs from scorpions: modifications and structure-activity. Front. Mol. Biosci. 9:887763. doi: 10.3389/fmolb.2022.887763, 35712354 PMC9197468

[ref5] Azeredo J. Azevedo N. F. Briandet R. Cerca N. Coenye T. Costa A. R. . (2017). Critical review on biofilm methods. Crit. Rev. Microbiol. 43, 313–351. doi: 10.1080/1040841X.2016.1208146, 27868469

[ref6] Azuama O. C. Ortiz S. Quirós-Guerrero L. Bouffartigues E. Tortuel D. Maillot O. . (2020). Tackling *Pseudomonas aeruginosa* virulence by Mulinane-like Diterpenoids from Azorella atacamensis. Biomolecules 10:1626. doi: 10.3390/biom10121626, 33276611 PMC7761567

[ref7] Bax A. Davis D. G. (1985). MLEV-17-based two-dimensional homonuclear magnetization transfer spectroscopy. J. Magnet. Res. 65, 355–360. doi: 10.1016/0022-2364(85)90018-6

[ref8] Ben Hur D. Kapach G. Wani N. A. Kiper E. Ashkenazi M. Smollan G. . (2022). Antimicrobial peptides against multidrug-resistant *Pseudomonas aeruginosa* biofilm from cystic fibrosis patients. J. Med. Chem. 65, 9050–9062. doi: 10.1021/acs.jmedchem.2c00270, 35759644 PMC9289885

[ref9] Blanka A. Schulz S. Eckweiler D. Franke R. Bielecka A. Nicolai T. . (2014). Identification of the alternative sigma factor SigX regulon and its implications for *Pseudomonas aeruginosa* pathogenicity. J. Bacteriol. 196, 345–356. doi: 10.1128/JB.01034-13, 24187091 PMC3911239

[ref10] Boechat A. L. Kaihami G. H. Politi M. J. Lépine F. Baldini R. L. (2013). A novel role for an ECF sigma factor in fatty acid biosynthesis and membrane fluidity in *Pseudomonas aeruginosa*. PLoS One 8:e84775. doi: 10.1371/journal.pone.0084775, 24386415 PMC3875570

[ref11] Cabot G. Zamorano L. Moyà B. Juan C. Navas A. Blázquez J. . (2016). Evolution of *Pseudomonas aeruginosa* antimicrobial resistance and fitness under low and high mutation rates. Antimicrob. Agents Chemother. 60, 1767–1778. doi: 10.1128/AAC.02676-15, 26729493 PMC4775977

[ref12] Castillo-Juárez I. Blancas-Luciano B. E. García-Contreras R. Fernández-Presas A. M. (2022). Antimicrobial peptides properties beyond growth inhibition and bacterial killing. PeerJ 10:e12667. doi: 10.7717/peerj.12667, 35116194 PMC8785659

[ref13] Chandler C. E. Horspool A. M. Hill P. J. Wozniak D. J. Schertzer J. W. Rasko D. A. . (2019). Genomic and phenotypic diversity among ten Laboratory isolates of *Pseudomonas aeruginosa* PAO1. J. Bacteriol. 201:18. doi: 10.1128/JB.00595-18, 30530517 PMC6379574

[ref71] Chan W. C. White P. D. (2000). Fmoc Solid Phase Peptide Synthesis: A Practical Approach. Oxford University Press.

[ref14] Cherny K. E. Sauer K. (2020). Untethering and degradation of the polysaccharide matrix are essential steps in the dispersion response of *Pseudomonas aeruginosa* biofilms. J. Bacteriol. 202:19. doi: 10.1128/JB.00575-19, 31712279 PMC6964737

[ref15] Chevalier S. Bouffartigues E. Bazire A. Tahrioui A. Duchesne R. Tortuel D. . (2019). Extracytoplasmic function sigma factors in *Pseudomonas aeruginosa*. Biochim. Biophys. Acta 1862, 706–721. doi: 10.1016/j.bbagrm.2018.04.008, 29729420

[ref72] Chevalier S. Bouffartigues E. Tortuel D. David A. Tahrioui A. Labbé C. . (2022). Cell Envelope Stress Response in Pseudomonas aeruginosa. Advances in experimental medicine and biology, 1386, 147–184. doi: 10.1007/978-3-031-08491-1_6, 36258072

[ref16] Chua S. L. Liu Y. Yam J. K. H. Chen Y. Vejborg R. M. Tan B. G. C. . (2014). Dispersed cells represent a distinct stage in the transition from bacterial biofilm to planktonic lifestyles. Nat. Commun. 5:4462. doi: 10.1038/ncomms5462, 25042103

[ref17] Ciofu O. Moser C. Jensen P. Ø. Høiby N. (2022). Tolerance and resistance of microbial biofilms. Nat. Rev. Microbiol. 20, 621–635. doi: 10.1038/s41579-022-00682-4, 35115704

[ref18] Ciofu O. Tolker-Nielsen T. (2019). Tolerance and resistance of *Pseudomonas aeruginosa* biofilms to antimicrobial agents—how *P. aeruginosa* can escape antibiotics. Front. Microbiol. 10:913. doi: 10.3389/fmicb.2019.00913, 31130925 PMC6509751

[ref19] Crivello G. Fracchia L. Ciardelli G. Boffito M. Mattu C. (2023). In vitro models of bacterial biofilms: innovative tools to improve understanding and treatment of infections. Nano 13:904. doi: 10.3390/nano13050904, 36903781 PMC10004855

[ref20] Daniele-Silva A. Machado R. J. A. Monteiro N. K. V. Estrela A. B. Santos E. C. G. Carvalho E. . (2016). Stigmurin and TsAP-2 from Tityus stigmurus scorpion venom: assessment of structure and therapeutic potential in experimental sepsis. Toxicon 121, 10–21. doi: 10.1016/j.toxicon.2016.08.016, 27567704

[ref21] Daniele-Silva A. Rodrigues S. D. C. S. Dos Santos E. C. G. Queiroz Neto M. F. D. Rocha H. A. D. O. Silva-Júnior A. A. D. . (2021). NMR three-dimensional structure of the cationic peptide Stigmurin from Tityus stigmurus scorpion venom: in vitro antioxidant and in vivo antibacterial and healing activity. Peptides 137:170478. doi: 10.1016/j.peptides.2020.170478, 33359395

[ref22] David A. Louis M. Tahrioui A. Rodrigues S. Labbé C. Maillot O. . (2024). cmpX overexpression in *Pseudomonas aeruginosa* affects biofilm formation and cell morphology in response to shear stress. Biofilm 7:100191. doi: 10.1016/j.bioflm.2024.100191, 38544741 PMC10965496

[ref23] de la Fuente-Núñez C. Reffuveille F. Fernández L. Hancock R. E. (2013). Bacterial biofilm development as a multicellular adaptation: antibiotic resistance and new therapeutic strategies. Curr. Opin. Microbiol. 16, 580–589. doi: 10.1016/j.mib.2013.06.013, 23880136

[ref24] De Melo E. T. Estrela A. B. Santos E. C. G. Machado P. R. L. Farias K. J. S. Torres T. M. . (2015). Structural characterization of a novel peptide with antimicrobial activity from the venom gland of the scorpion Tityus stigmurus: Stigmurin. Peptides 68, 3–10. doi: 10.1016/j.peptides.2015.03.003, 25805002

[ref25] Diggle S. P. Stacey R. E. Dodd C. Cámara M. Williams P. Winzer K. (2006). The galactophilic lectin, LecA, contributes to biofilm development in *Pseudomonas aeruginosa*. Environ. Microbiol. 8, 1095–1104. doi: 10.1111/j.1462-2920.2006.001001.x, 16689730

[ref26] Fléchard M. Duchesne R. Tahrioui A. Bouffartigues E. Depayras S. Hardouin J. . (2018). The absence of SigX results in impaired carbon metabolism and membrane fluidity in *Pseudomonas aeruginosa*. Sci. Rep. 8:17212. doi: 10.1038/s41598-018-35503-3, 30464317 PMC6249292

[ref27] Flemming H.-C. Wingender J. (2010). The biofilm matrix. Nat. Rev. Microbiol. 8, 623–633. doi: 10.1038/nrmicro2415, 20676145

[ref28] Flemming H.-C. Wingender J. Szewzyk U. Steinberg P. Rice S. A. Kjelleberg S. (2016). Biofilms: an emergent form of bacterial life. Nat. Rev. Microbiol. 14, 563–575. doi: 10.1038/nrmicro.2016.94, 27510863

[ref29] Furtado A. A. Daniele-Silva A. de Oliveira I. R. R. Mendes R. F. V. Santos E. C. G. Carvalho E. . (2022). In silico and in vitro structure-stability-function relationship of analog peptides of Stigmurin and its antibacterial and antibiofilm activities. Pharmacol. Res. 181:106245. doi: 10.1016/j.phrs.2022.106245, 35526666

[ref30] Furtado A. A. Daniele-Silva A. Silva-Júnior A. A. D. Fernandes-Pedrosa M. D. F. (2020). Biology, venom composition, and scorpionism induced by brazilian scorpion Tityus stigmurus (Thorell, 1876) (Scorpiones: Buthidae): a mini-review. Toxicon 185, 36–45. doi: 10.1016/j.toxicon.2020.06.015, 32585220

[ref31] Gautier R. Douguet D. Antonny B. Drin G. (2008). HELIQUEST: a web server to screen sequences with specific α-helical properties. Bioinformatics 24, 2101–2102. doi: 10.1093/bioinformatics/btn392, 18662927

[ref32] Gonçalves T. Vasconcelos U. (2021). Colour me blue: the history and the biotechnological potential of Pyocyanin. Molecules 26:927. doi: 10.3390/molecules26040927, 33578646 PMC7916356

[ref33] Guilhen C. Forestier C. Balestrino D. (2017). Biofilm dispersal: multiple elaborate strategies for dissemination of bacteria with unique properties. Mol. Microbiol. 105, 188–210. doi: 10.1111/mmi.13698, 28422332

[ref34] Han A. Lee S.-Y. (2023). An overview of various methods for in vitro biofilm formation: a review. Food Sci. Biotechnol. 32, 1617–1629. doi: 10.1007/s10068-023-01425-8, 37780598 PMC10533769

[ref35] Hancock R. E. W. Scott M. G. (2000). The role of antimicrobial peptides in animal defenses. Proc. Natl. Acad. Sci. 97, 8856–8861. doi: 10.1073/pnas.97.16.8856, 10922046 PMC34023

[ref36] Heydorn A. Nielsen A. T. Hentzer M. Sternberg C. Givskov M. Ersbøll B. K. . (2000). Quantification of biofilm structures by the novel computer program comstat. Microbiology 146, 2395–2407. doi: 10.1099/00221287-146-10-2395, 11021916

[ref37] Hyberts S. G. Goldberg M. S. Havel T. F. Wagner G. (1992). The solution structure of eglin c based on measurements of many NOEs and coupling constants and its comparison with X-ray structures. Protein Sci. 1, 736–751. doi: 10.1002/pro.5560010606, 1304915 PMC2142248

[ref38] Jennings L. K. Storek K. M. Ledvina H. E. Coulon C. Marmont L. S. Sadovskaya I. . (2015). Pel is a cationic exopolysaccharide that cross-links extracellular DNA in the *Pseudomonas aeruginosa* biofilm matrix. Proc. Natl. Acad. Sci. 112, 11353–11358. doi: 10.1073/pnas.1503058112, 26311845 PMC4568648

[ref39] Johnson B. A. Blevins R. A. (1994). NMR view: a computer program for the visualization and analysis of NMR data. J. Biomol. NMR 4, 603–614. doi: 10.1007/BF00404272, 22911360

[ref40] Kolpen M. Kragh K. N. Enciso J. B. Faurholt-Jepsen D. Lindegaard B. Egelund G. B. . (2022). Bacterial biofilms predominate in both acute and chronic human lung infections. Thorax 77, 1015–1022. doi: 10.1136/thoraxjnl-2021-217576, 35017313 PMC9510407

[ref41] Kragh K. N. Tolker-Nielsen T. Lichtenberg M. (2023). The non-attached biofilm aggregate. Commun. Biol. 6:898. doi: 10.1038/s42003-023-05281-4, 37658117 PMC10474055

[ref42] Kumar A. Ernst R. R. Wüthrich K. (1980). A two-dimensional nuclear Overhauser enhancement (2D NOE) experiment for the elucidation of complete proton-proton cross-relaxation networks in biological macromolecules. Biochem. Biophys. Res. Commun. 95, 1–6. doi: 10.1016/0006-291X(80)90695-6, 7417242

[ref43] Letizia M. Diggle S. P. Whiteley M. (2025). *Pseudomonas aeruginosa*: ecology, evolution, pathogenesis and antimicrobial susceptibility. Nat. Rev. Microbiol. 23, 701–717. doi: 10.1038/s41579-025-01193-8, 40442328 PMC13064840

[ref44] Louis M. Clamens T. Tahrioui A. Desriac F. Rodrigues S. Rosay T. . (2022). *Pseudomonas aeruginosa* biofilm dispersion by the human atrial natriuretic peptide. Adv. Sci. 9:2103262. doi: 10.1002/advs.202103262, 35032112 PMC8895129

[ref45] Louis M. Tahrioui A. Tremlett C. J. Clamens T. Leprince J. Lefranc B. . (2023). The natriuretic peptide receptor agonist osteocrin disperses *Pseudomonas aeruginosa* biofilm. Biofilm 5:100131. doi: 10.1016/j.bioflm.2023.100131, 37252226 PMC10220261

[ref46] Moradali M. F. Ghods S. Rehm B. H. A. (2017). *Pseudomonas aeruginosa* lifestyle: a paradigm for adaptation, survival, and persistence. Front. Cell. Infect. Microbiol. 7:39. doi: 10.3389/fcimb.2017.00039, 28261568 PMC5310132

[ref47] Mulcahy L. R. Isabella V. M. Lewis K. (2014). *Pseudomonas aeruginosa* biofilms in disease. Microb. Ecol. 68, 1–12. doi: 10.1007/s00248-013-0297-x, 24096885 PMC3977026

[ref48] Pang Z. Raudonis R. Glick B. R. Lin T.-J. Cheng Z. (2019). Antibiotic resistance in *Pseudomonas aeruginosa*: mechanisms and alternative therapeutic strategies. Biotechnol. Adv. 37, 177–192. doi: 10.1016/j.biotechadv.2018.11.013, 30500353

[ref49] Parente A. M. S. Daniele-Silva A. Furtado A. A. Melo M. A. Lacerda A. F. Queiroz M. . (2018). Analogs of the scorpion venom peptide Stigmurin: structural assessment, toxicity, and increased antimicrobial activity. Toxins 10:161. doi: 10.3390/toxins10040161, 29670004 PMC5923327

[ref50] Passos Da Silva D. Matwichuk M. L. Townsend D. O. Reichhardt C. Lamba D. Wozniak D. J. . (2019). The *Pseudomonas aeruginosa* lectin LecB binds to the exopolysaccharide Psl and stabilizes the biofilm matrix. Nat. Commun. 10:2183. doi: 10.1038/s41467-019-10201-4, 31097723 PMC6522473

[ref51] Pollastri G. McLysaght A. (2005). Porter: a new, accurate server for protein secondary structure prediction. Bioinformatics 21, 1719–1720. doi: 10.1093/bioinformatics/bti203, 15585524

[ref52] Qin S. Xiao W. Zhou C. Pu Q. Deng X. Lan L. . (2022). *Pseudomonas aeruginosa*: pathogenesis, virulence factors, antibiotic resistance, interaction with host, technology advances and emerging therapeutics. Signal Transduct. Target. Ther. 7:199. doi: 10.1038/s41392-022-01056-1, 35752612 PMC9233671

[ref53] Rahmani-Badi A. Sepehr S. Babaie-Naiej H. (2015). A combination of cis-2-decenoic acid and chlorhexidine removes dental plaque. Arch. Oral Biol. 60, 1655–1661. doi: 10.1016/j.archoralbio.2015.08.006, 26351749

[ref54] Rangel K. Curty Lechuga G. Almeida Souza A. L. Rangel Da Silva Carvalho J. P. Simões Villas Bôas M. H. De Simone S. G. (2020). Pan-drug resistant *Acinetobacter baumannii*, but not other strains, are resistant to the bee venom peptide Melittin. Antibiotics 9:178. doi: 10.3390/antibiotics9040178, 32295149 PMC7235889

[ref55] Rumbaugh K. P. Sauer K. (2020). Biofilm dispersion. Nat. Rev. Microbiol. 18, 571–586. doi: 10.1038/s41579-020-0385-0, 32533131 PMC8564779

[ref56] Schanda P. Kupče Ē. Brutscher B. (2005). SOFAST-HMQC experiments for recording two-dimensional Deteronuclear correlation spectra of proteins within a few seconds. J. Biomol. NMR 33, 199–211. doi: 10.1007/s10858-005-4425-x, 16341750

[ref57] Schwartz C. F. A. Lerche C. J. Svanekjær T. Kragh K. N. Laulund A. S. Thomsen K. . (2020). In vivo demonstration of *Pseudomonas aeruginosa* biofilms as independent pharmacological microcompartments. J. Cyst. Fibros. 19, 996–1003. doi: 10.1016/j.jcf.2020.01.009, 32067957

[ref58] Tahrioui A. Ortiz S. Azuama O. C. Bouffartigues E. Benalia N. Tortuel D. . (2020). Membrane-interactive compounds from *Pistacia lentiscus* L. thwart *Pseudomonas aeruginosa* virulence. Front. Microbiol. 11:1068. doi: 10.3389/fmicb.2020.01068, 32528451 PMC7264755

[ref59] Tareau A.-S. Tahrioui A. Gonzalez M. Croize E. Varin Simon J. Barreau M. . (2025). Squalamine and claramine A1 disperse *Pseudomonas aeruginosa* biofilm. Biofilm 9:100293. doi: 10.1016/j.bioflm.2025.100293, 40519941 PMC12166708

[ref60] Thakur A. Alajangi H. K. Sharma A. Hwang E. Khajuria A. Kumari L. . (2025). Stigmurin encapsulated PLA–PEG ameliorates its therapeutic potential, antimicrobial and antiproliferative activities. Discover Nano 20:50. doi: 10.1186/s11671-025-04224-8, 40063147 PMC11893973

[ref61] Tolker-Nielsen T. Sternberg C. (2014). “Methods for studying biofilm formation: flow cells and confocal laser scanning microscopy,” in Pseudomonas Methods and Protocols, eds. Filloux A. Ramos J.-L. (New York: Springer), 615–629. 10.1007/978-1-4939-0473-0_47 24818937

[ref62] Uruén C. Chopo-Escuin G. Tommassen J. Mainar-Jaime R. C. Arenas J. (2020). Biofilms as promoters of bacterial antibiotic resistance and tolerance. Antibiotics 10:3. doi: 10.3390/antibiotics10010003, 33374551 PMC7822488

[ref73] Uzair B. Bint-e-Irshad S. Khan B. A. Azad B. Mahmood T. Rehman M. U. . (2018). Scorpion venom peptides as a potential source for human drug candidates. Protein & Peptide Letters, 25, 702–708. doi: 10.2174/0929866525666180614114307, 29921194

[ref63] Willker W. Leibfritz D. Kerssebaum R. Bermel W. (1993). Gradient selection in inverse heteronuclear correlation spectroscopy. Magn. Reson. Chem. 31, 287–292. doi: 10.1002/mrc.1260310315

[ref64] Xia Z. Xie L. Li B. Lv X. Zhang H. Cao Z. (2024). Antimicrobial potential of scorpion-venom-derived peptides. Molecules 29:5080. doi: 10.3390/molecules29215080, 39519721 PMC11547508

