# Supplementary material for: Stigmurin derivatives as potent-biofilm eradicating agents against the major human opportunistic pathogen Pseudomonas aeruginosa
Source: Front Microbiol. 2026 May 28;17:1827386. doi: 10.3389/fmicb.2026.1827386 (PMC13253951; doi:10.3389/fmicb.2026.1827386)
Supplement: Supplementary file 1 [file Data_Sheet_1.docx]

**Supplementary material**

**Stigmurin derivatives as potent-biofilm eradicating agents against the major human opportunistic pathogen *Pseudomonas aeruginosa***

Anne Sophie Tareau^1,2^, Adriana Marina e Silva Parente^3^, Allanny Alves Furtado^3^, Magalie Barreau^1,2^, Hung Le^1,2^, Bruno Amorim-Carmo^3^, Lucas Hilário Nogueira de Sousa^4^, Olivier Maillot^1,2^, Mathieu Gonzalez^1,2^, Adrien Forge^1,2^, Ali Tahrioui^1,2^, Jarbas Magalhaes Resende^5^, Olivier Lesouhaitier^1,2^, Renata Mendonça Araújo^4^, Matheus de Freitas Fernandes-Pedrosa^3^, & Sylvie Chevalier^1,2^*

**Supplementary Tables**

**Table S1** **–** Chemical shifts of amino acids (A.A.) residues in StigA31 determined in TFE-*d_2_*:H_2_O (40:60%, v:v).

| **A.A.** | **H** | **H_α_** | **H_β_** | **H_γ_** | **H_δ_** | **H_ε_** | **H_ζ_** | **NHT** | **C_α_** | **C_β_** | **C_γ_** | **C_δ_** | **C_ε_** |
| --- | --- | --- | --- | --- | --- | --- | --- | --- | --- | --- | --- | --- | --- |
| 1 F |  | 4.18 | 3.15 |  | 7.24 | 7.34 |  |  | 57.47 | 39.61 |  | 57.20 | 56.62 |
|  |  |  | 3.15 |  |  |  |  |  |  |  |  |  |  |
| 2 F | 8.04 | 4.61 | 3.02 |  | 7.16 | 7.19 |  |  | 57.78 | 39.89 |  | 56.32 | 54.27 |
|  |  |  | 2.97 |  |  |  |  |  |  |  |  |  |  |
| 3 K | 7.91 | 4.17 | 1.73 | 1.37 | 1.64 | 2.93 |  |  | 57.20 | 33.45 | 24.69 | 29.34 | 42.13 |
|  |  |  | 1.64 | 1.31 | 1.64 | 2.93 |  |  |  |  |  |  |  |
| 4 L | 7.47 | 4.25 | 1.52 | 1.51 | 0.84 |  |  |  | 55.01 | 42.55 | 27.01 | 24.07 |  |
|  |  |  | 1.48 |  | 0.89 |  |  |  |  |  |  | 24.43 |  |
| 5 I | 7.39 | 4.32 | 1.97 | 1.53 | 0.87 |  |  |  | 61.04 | 37.56 | 27.58 | 12.05 |  |
|  |  |  |  | 0.93 |  |  |  |  |  |  | 17.24 |  |  |
|  |  |  |  | 1.18 |  |  |  |  |  |  |  |  |  |
| 6 P |  | 4.17 | 1.89 | 1.93 | 3.71 |  |  |  | 65.70 | 31.69 | 27.59 | 50.41 |  |
|  |  |  | 2.35 | 2.12 | 3.82 |  |  |  |  |  |  |  |  |
| 7 K | 7.81 | 3.95 | 1.83 | 1.40 | 1.66 | 2.90 |  |  | 62.04 | 32.29 | 25.23 | 28.78 | 42.11 |
|  |  |  | 1.87 | 1.52 | 1.66 | 2.90 |  |  |  |  |  |  |  |
| 8 L | 7.63 | 4.16 | 1.73 | 1.61 | 0.87 |  |  |  | 56.56 | 42.25 | 27.01 | 24.29 |  |
|  |  |  | 1.68 |  | 0.92 |  |  |  |  |  |  | 23.52 |  |
| 9 V | 7.80 | 3.54 | 2.08 | 0.98 |  |  |  |  | 66.89 | 31.69 | 20.56 |  |  |
|  |  |  |  | 0.91 |  |  |  |  |  |  | 22.31 |  |  |
| 10 K | 7.81 | 3.88 | 1.86 | 1.37 | 1.64 | 2.94 | 7.63 |  | 60.14 | 32.58 | 25.82 | 29.10 | 41.86 |
|  |  |  | 1.78 | 1.59 | 1.64 | 2.94 |  |  |  |  |  |  |  |
| 11 K | 7.52 | 3.97 | 1.98 | 1.60 | 1.67 | 2.93 |  |  | 59.55 | 32.31 | 24.95 | 29.35 | 41.96 |
|  |  |  | 2.00 | 1.39 | 1.67 | 2.93 |  |  |  |  |  |  |  |
| 12 L | 8.14 | 4.03 | 1.50 | 1.76 | 0.91 |  |  |  | 58.06 | 41.67 | 26.71 | 23.49 |  |
|  |  |  | 1.92 |  | 0.82 |  |  |  |  |  |  | 22.32 |  |
| 13 I | 8.44 | 3.69 | 1.88 | 1.75 | 0.78 |  |  |  | 65.11 | 37.84 | 28.47 | 12.64 |  |
|  |  |  |  | 1.14 |  |  |  |  |  |  | 16.75 |  |  |
|  |  |  |  | 0.88 |  |  |  |  |  |  |  |  |  |
| 14 K | 7.79 | 3.97 | 1.91 | 1.43 | 1.66 | 2.95 | 8.14 |  | 58.99 | 32.28 | 24.96 | 30.22 | 42.20 |
|  |  |  | 1.93 | 1.54 | 1.66 | 2.95 |  |  |  |  |  |  |  |
| 15 A | 7.91 | 4.09 | 1.33 |  |  |  |  |  | 54.27 | 18.06 |  |  |  |
| 16 F | 8.26 | 4.53 | 3.12 |  | 7.26 | 7.32 |  |  | 58.98 | 39.33 |  | 55.75 | 55.74 |
|  |  |  | 3.19 |  |  |  |  |  |  |  |  |  |  |
| 17 K | 8.26 | 4.25 | 1.90 | 1.53 | 1.67 | 2.96 | 7.90 | 7.13 | 56.32 | 32.51 | 24.69 | 29.34 | 42.02 |
|  |  |  | 1.87 | 1.47 | 1.67 | 2.96 |  | 6.94 |  |  |  |  |  |

**Supplementary Figures**


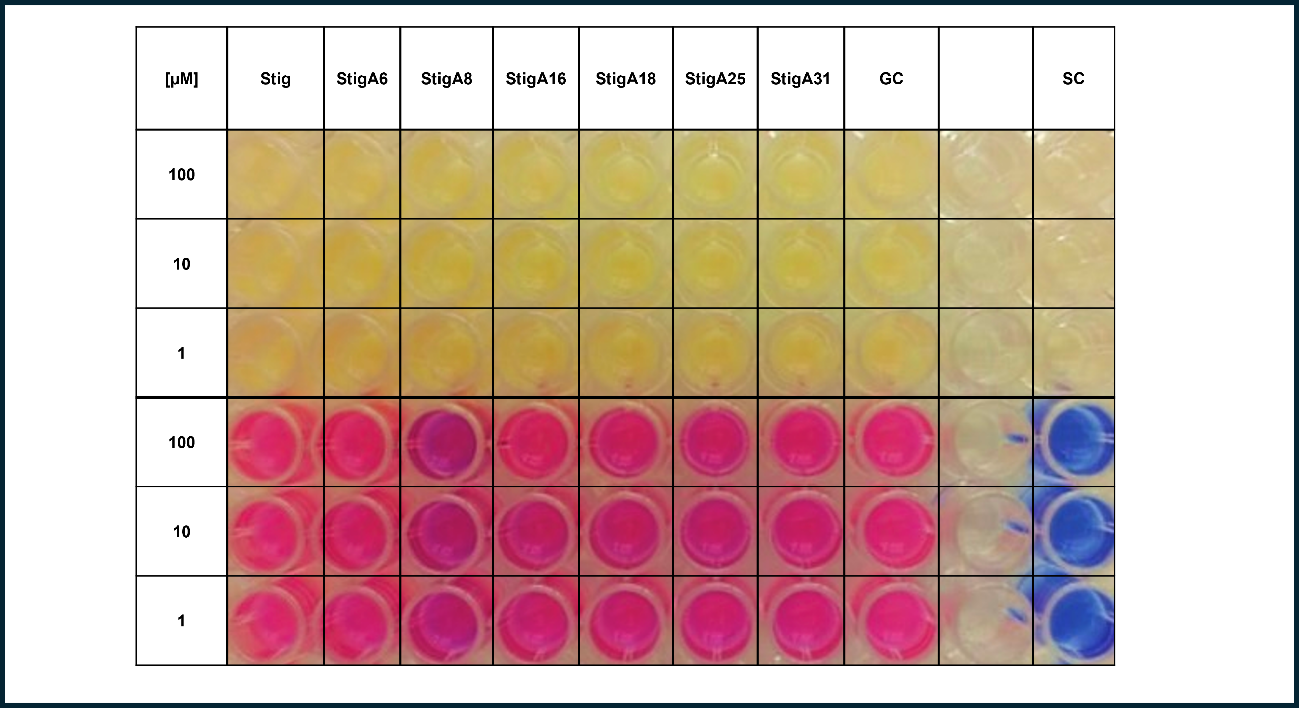


**Figure S1. Minimal inhibitory concentrations (MIC) of Stig and its derived StigA6, StigA8, StigA16, StigA18, StigA25 and StigA31 against *P. aeruginosa* H103.** Bacterial growth was observed in absence (upper part) or in presence of resazurin (lower part) to ascertain bacterial viability. Pink or purple color is the reflect of metabolically active cells. Blue color is the reflect of metabolically inactive or dead cells. GC: Growth control. SC: Sterile control.


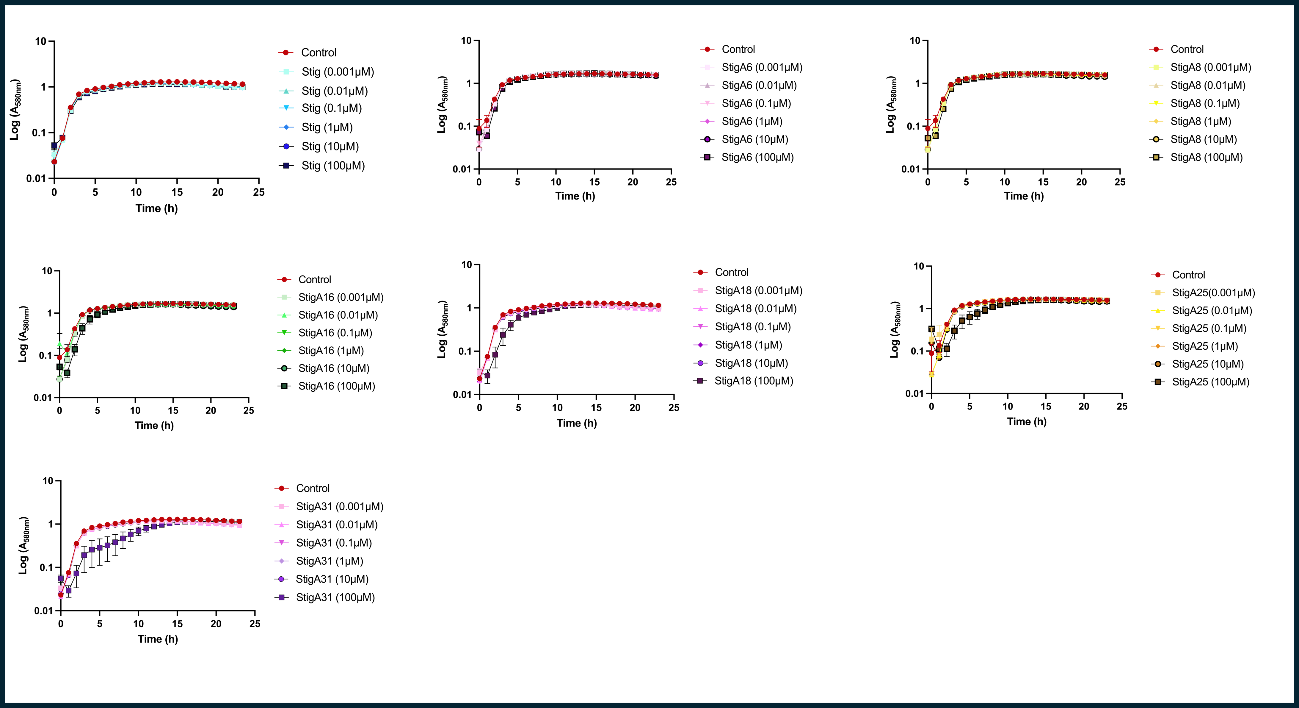


**Figure S2. Growth kinetics of *P. aeruginosa* H103 in presence of various concentrations of Stig and its analog peptides StigA6, StigA8, StigA16, StigA18, StigA25 and StigA31 ranging from 0.001 to 100 µM.** The error bars represent the standard error of the means (SEMs) and are the result of the analysis of three independent biological assays.

**
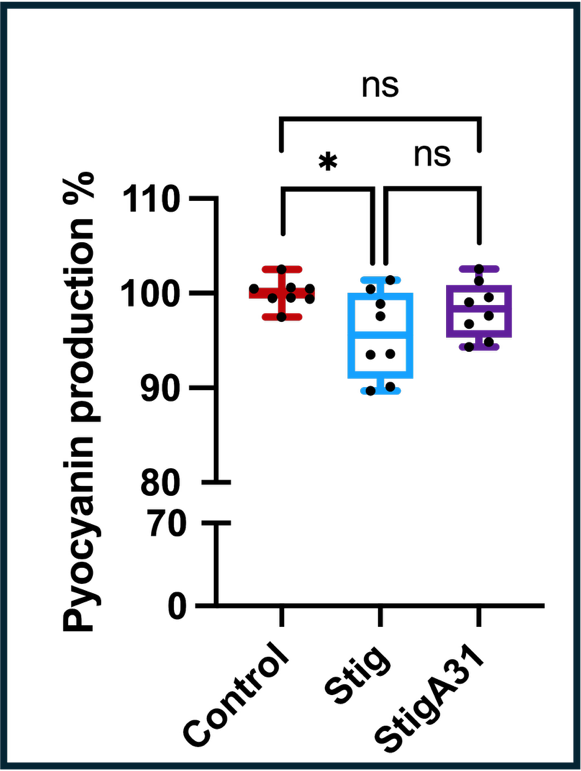
**

**Figure S3. Stig, but not StigA31, slightly decreased pyocyanin production.** *P. aeruginosa* H103 was treated with ultrapure Milli-Q^®^ water (red bar), Stig (blue bar), or StigA31 (purple bar). Each experiment was assayed four times independently. Statistics were performed by ordinary one‐way ANOVA followed by Tukey's multiple‐comparison test. Significance was considered at *, *p* = 0.01–0.05; ns (not significant), p > 0.05.

**
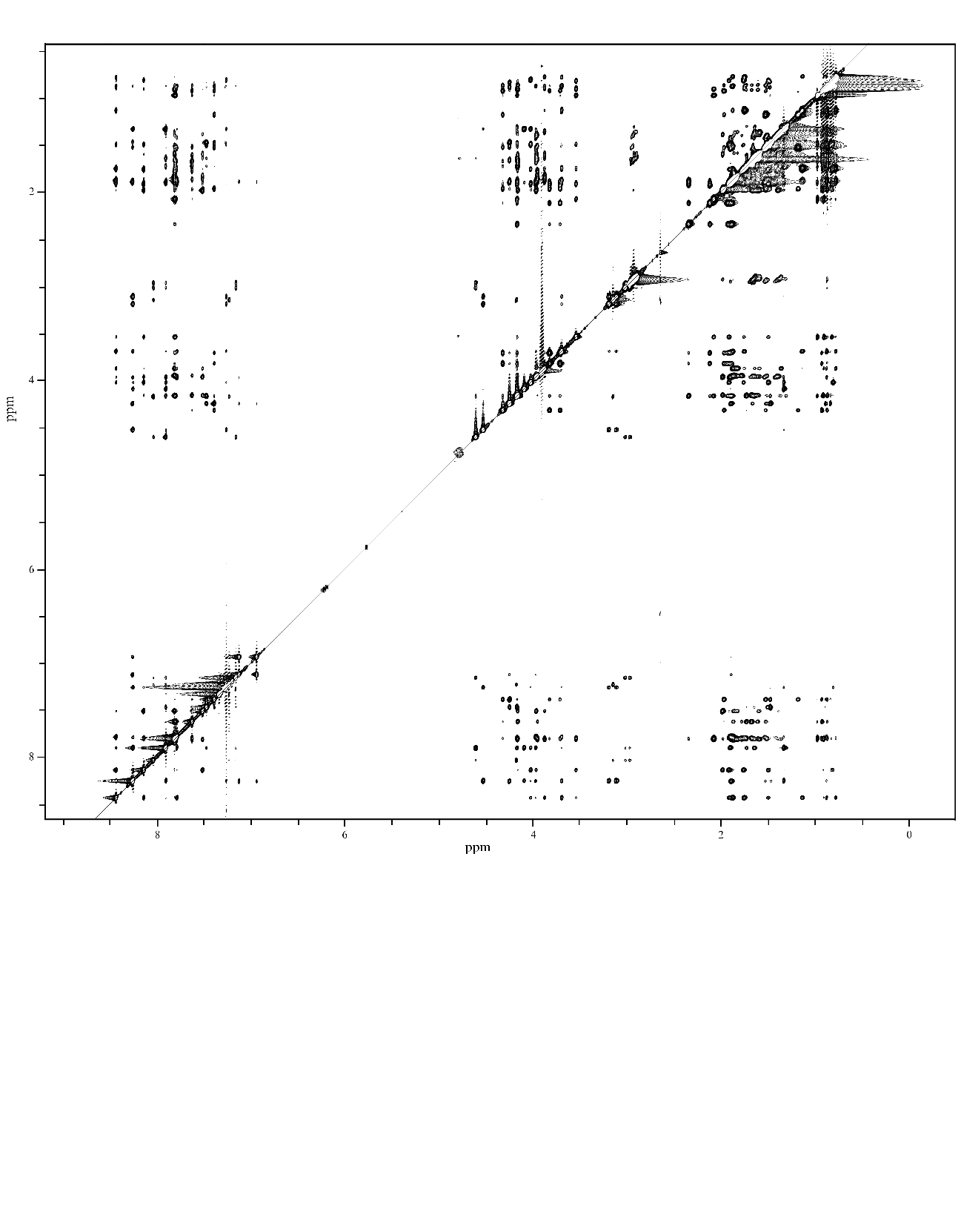
**

**Figure S4. ^1^H-^1^H NOESY spectrum of StigA31** in TFE-*d_2_*:H_2_O (40:60%, v:v), with chemical shift ranges of -0.56–9.53 ppm (X-axis) and 0.42–8.72 ppm (Y-axis).


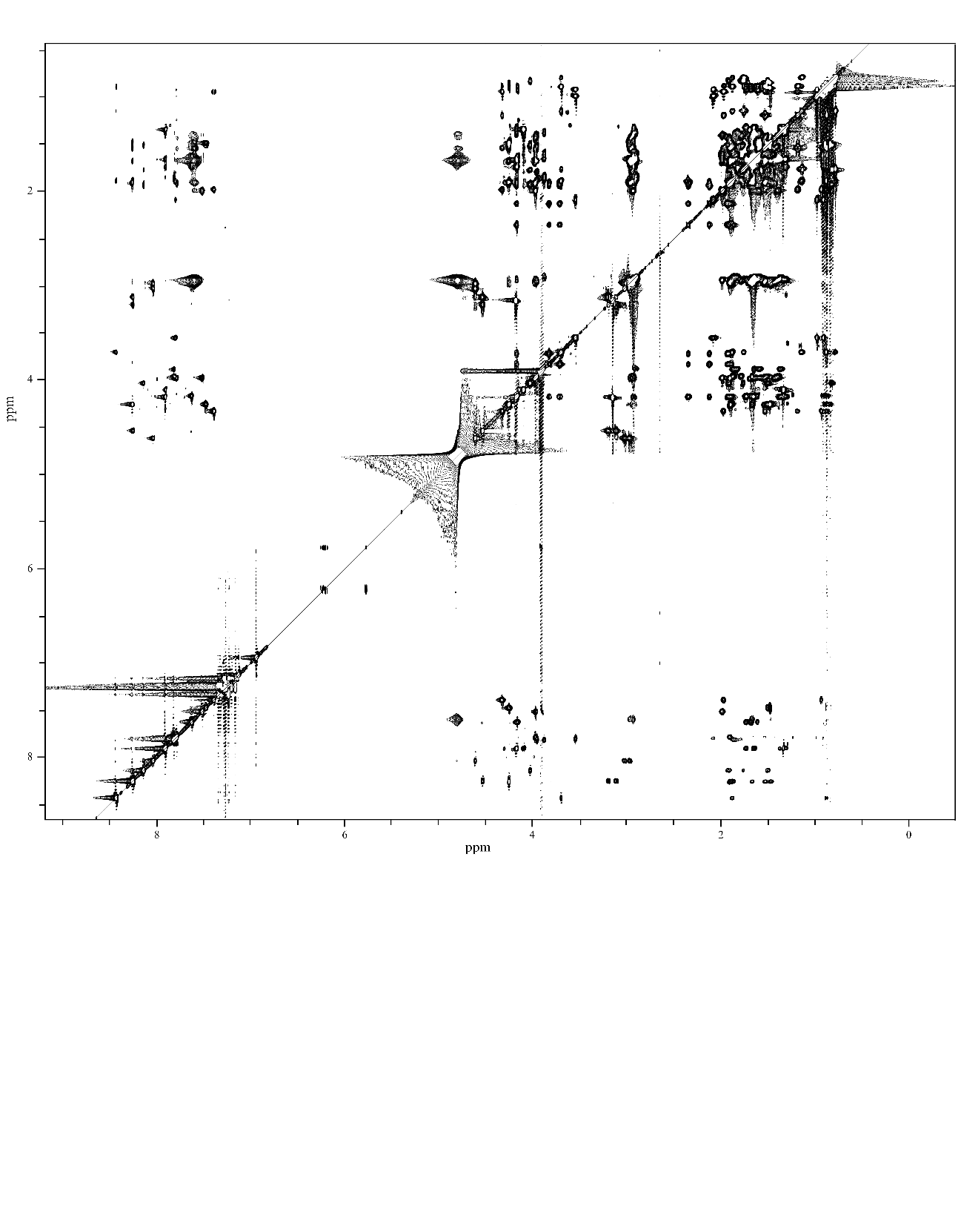


**Figure S5. ^1^H-^1^H TOCSY spectrum of StigA31** in TFE-*d_2_*:H_2_O (40:60%, v:v), with chemical shift ranges of -0.56–9.53 ppm (X-axis) and 0.42–8.72 ppm (Y-axis).


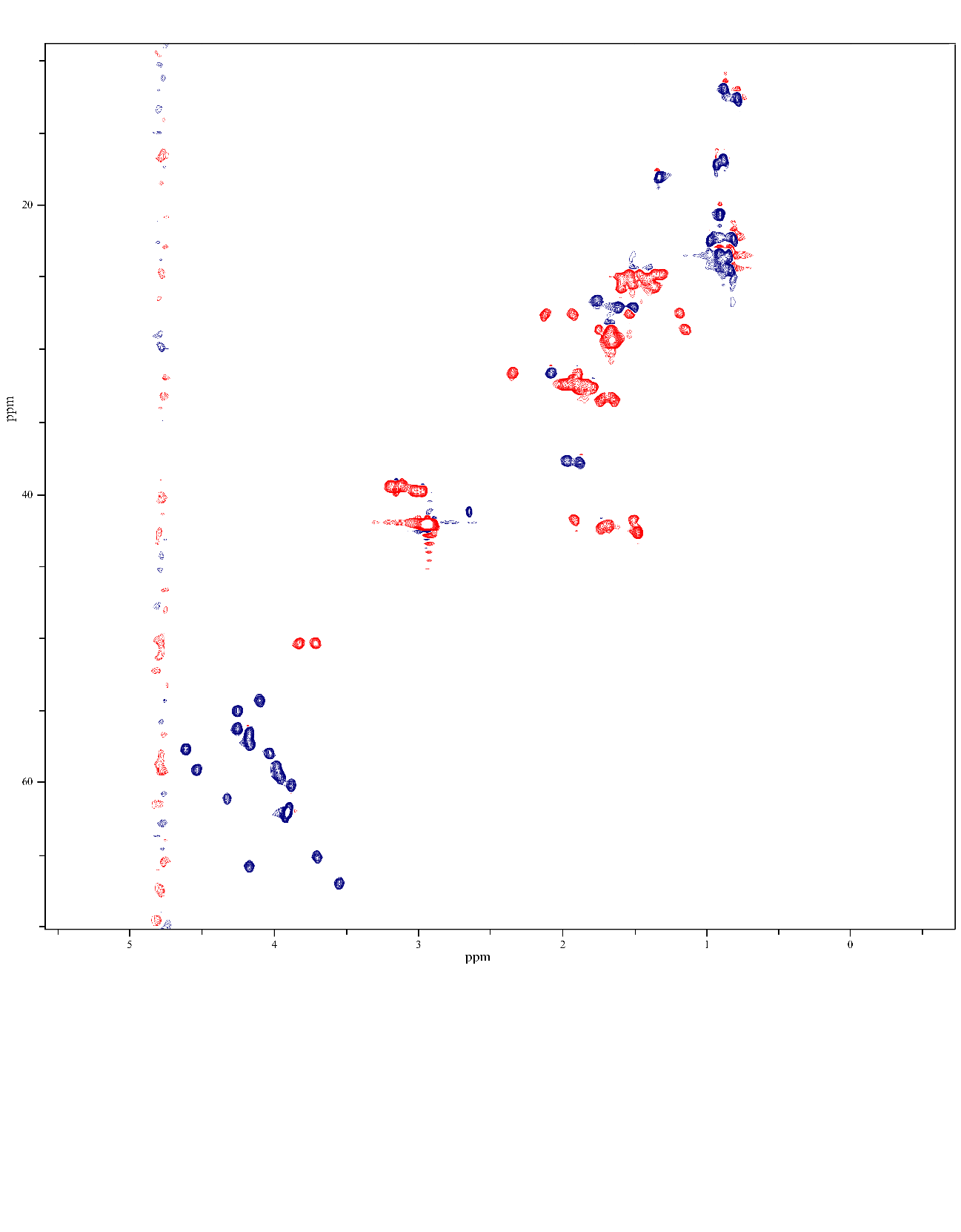


**Figure S6. ^1^H-^13^C–HSQC spectrum of StigA31** in TFE-*d_2_*:H_2_O (40:60%, v:v), with chemical shift ranges of -0.71–5.59 ppm (X-axis) and 8.85–70.12 ppm (Y-axis).

**
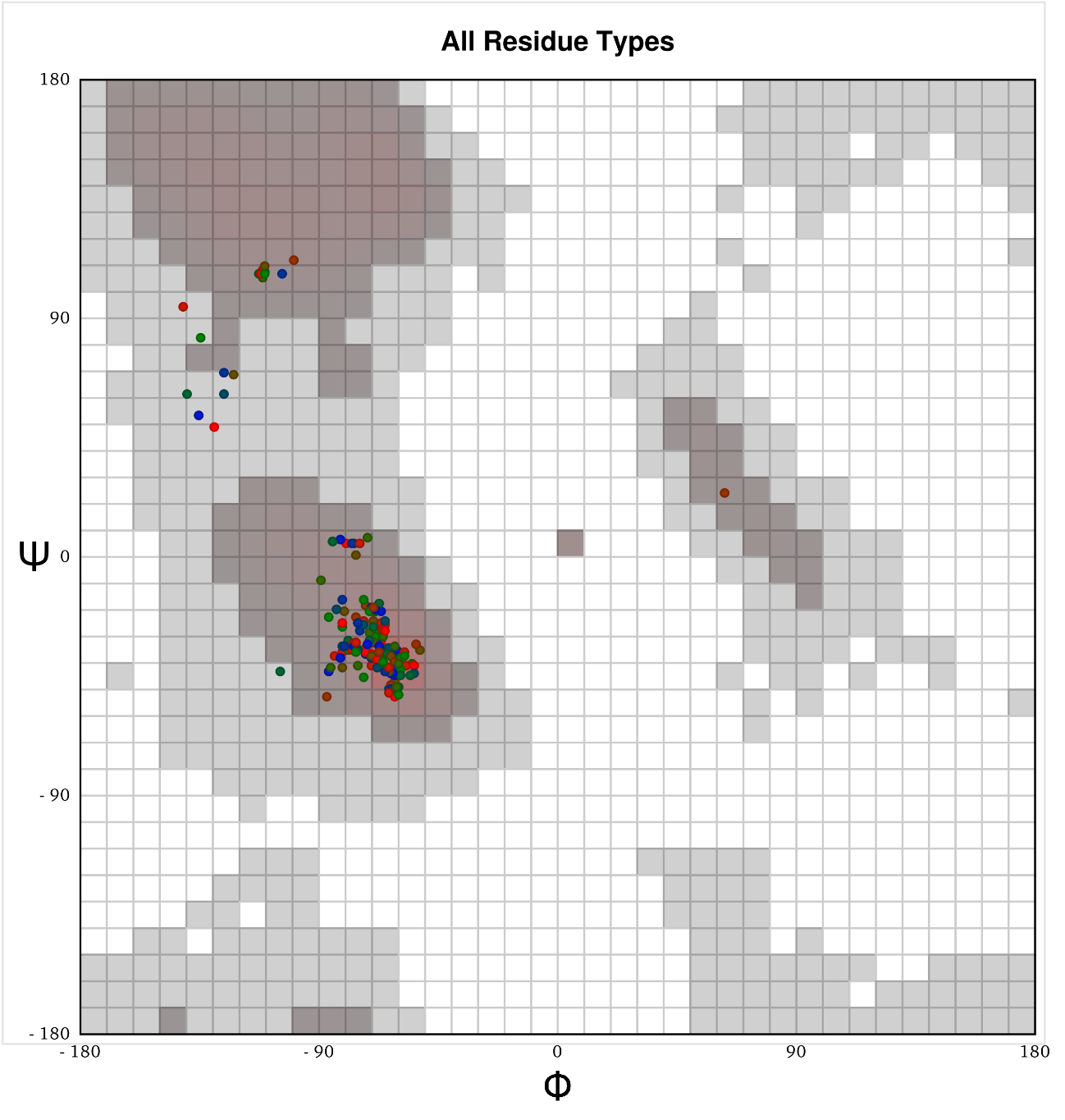
**

**Figure S7. Backbone Dihedral Angle Distribution** (Ramachandran Plot) of StigA31 in TFE-*d_2_*:H_2_O (40:60%, v:v).
